# Supplementary material for: The shared ancestry between the C9orf72 hexanucleotide repeat expansion and intermediate-length alleles using haplotype sharing trees and HAPTK
Source: Am J Hum Genet. 2024 Jan 18;111(2):383–92. doi: 10.1016/j.ajhg.2023.12.019 (PMC10870140; doi:10.1016/j.ajhg.2023.12.019)
Supplement: Document S1. Figures S1–S8 and supplemental methods [file mmc1.pdf]

The American Journal of Human Genetics, Volume *111*

Supplemental information

The shared ancestry between the *C9orf72* hexanucleotide repeat expansion and intermediate-length alleles using haplotype sharing trees and HAPTK

Osma S. Rautila, Karri Kaivola, Harri Rautila, Laura Hokkanen, Jyrki Launes, Timo E. Strandberg, Hannu Laaksovirta, Johanna Palmio, and Pentti J. Tienari

Supplemental Figures

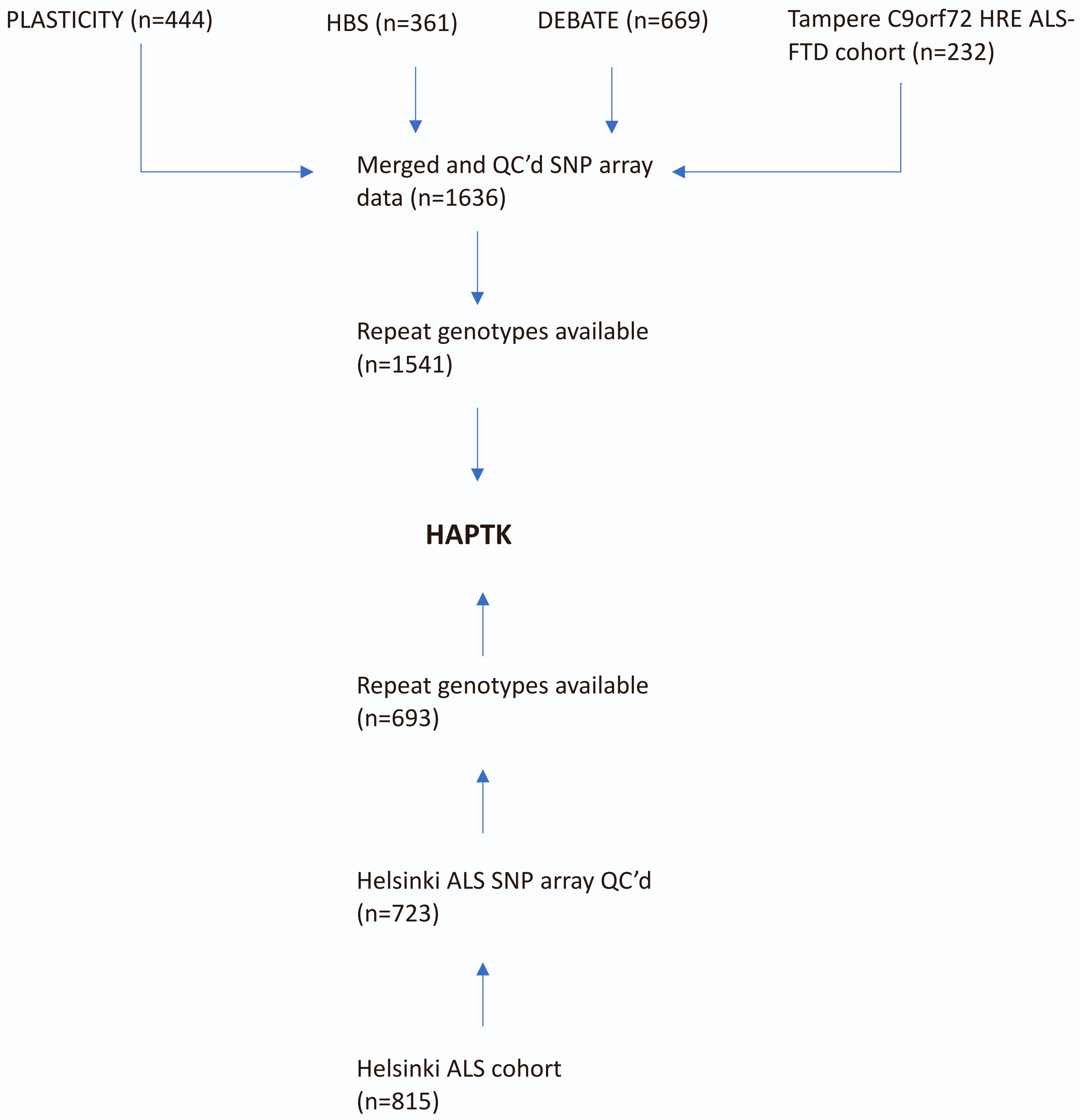

Figure S1. A chart of the study
Illumina GSA v2-3 chip cohorts on the top and the FinnGen Affymetrix Axiom Custom SNP array Helsinki ALS samples on the bottom. The number of samples per different repeat genotype group can be found in Tables S4 and S5.

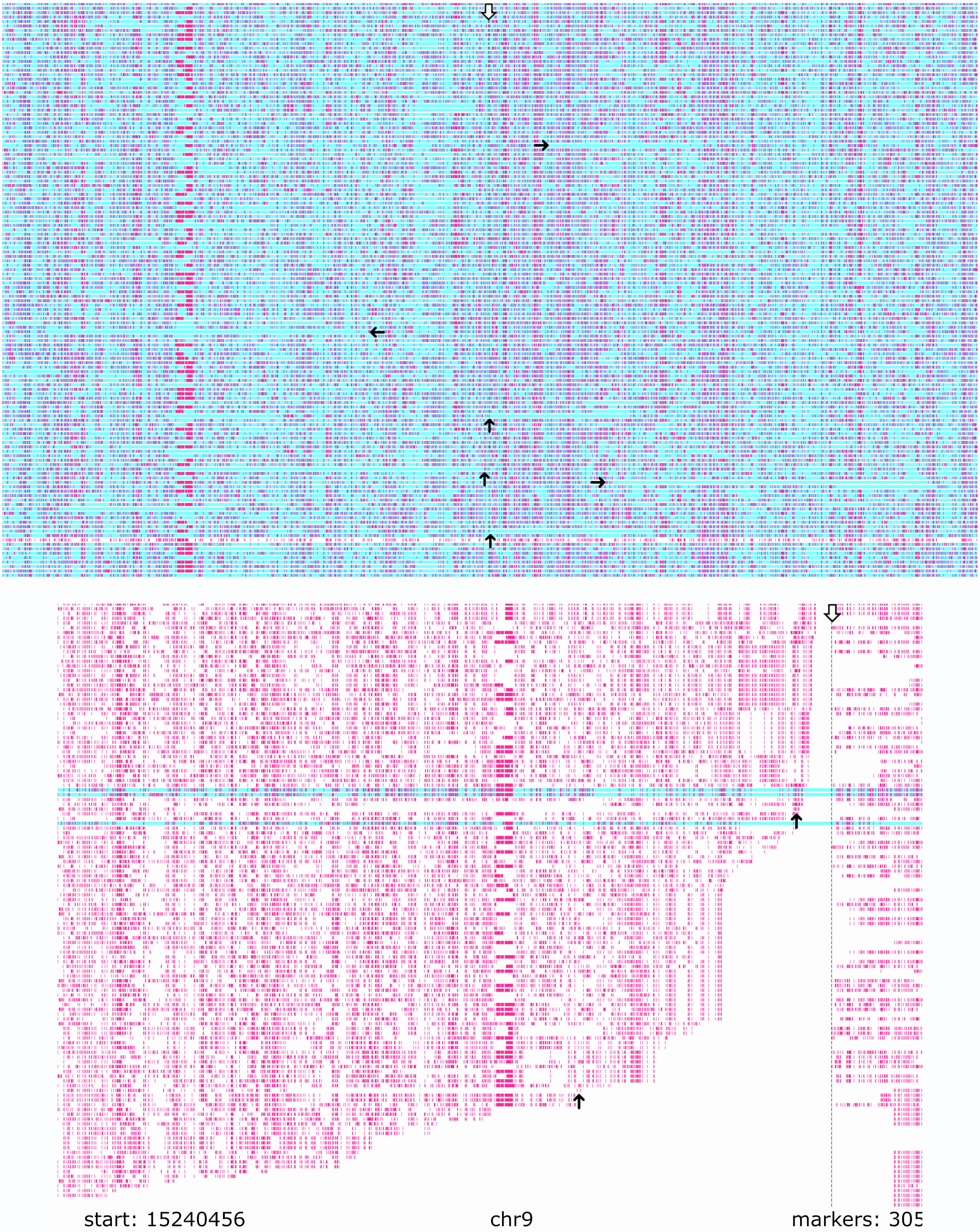

Figure S2. Switch-error and genotyping error evaluation based on the comparison graph
The white down-arrow on the top denotes the starting point of the algorithm (the C9orf72 HRE locus). The black up-arrows denote suspected genotyping errors or small switch-errors. Left and right arrows denote runs of ancestral sequence that are most likely due to a switch-error further away from the starting locus. The haplotypes tagged in blue were removed when selecting only for the longer ancestral sequence sharing haplotypes per sample.

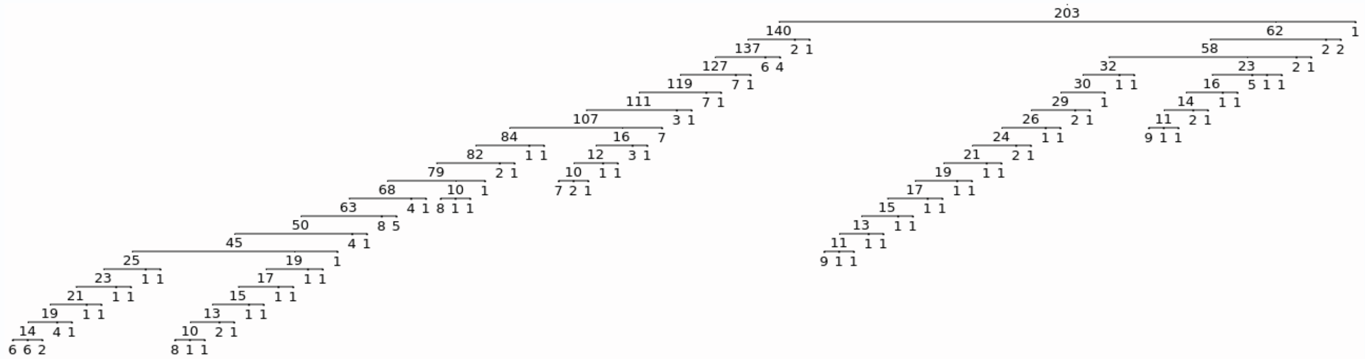

Figure S3. The Tampere ALS-FTD cohort bidirectional HST of HRE haplotypes
The cohort is genotyped on the Illumina GSA v3 SNP array. For visualization purposes the branching is cut after 10 samples.

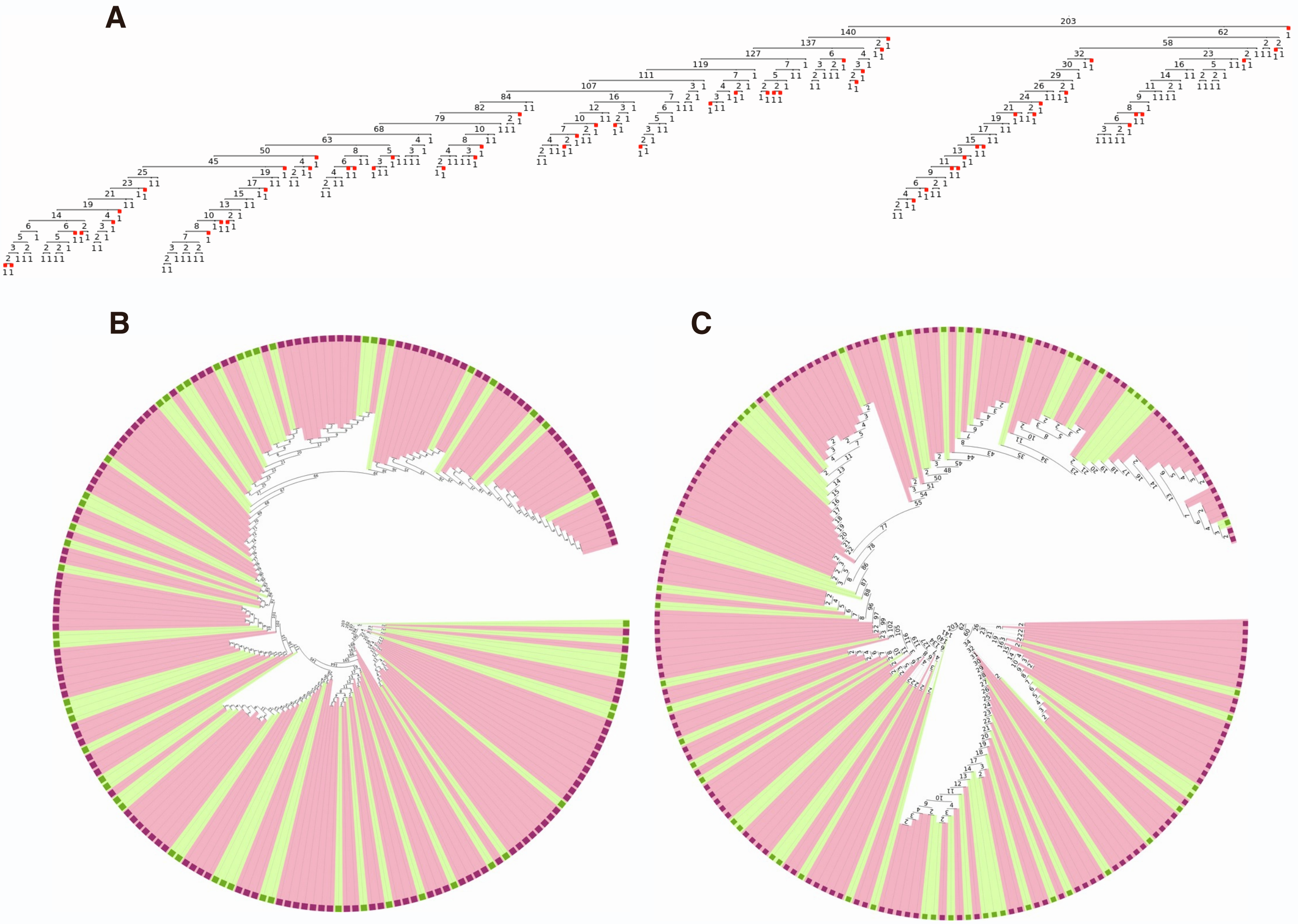

Figure S4. The Tampere ALS-FTD cohort bidirectional HST of HRE haplotypes with FTD samples tagged in red
(A, B, C) The FTD cases are relatively uniformly distributed along the tree and do not form their own subtrees.
(A) The bidirectional HST with FTD samples marked in red. No circular layout.
(B) The left side unidirectional HST with FTD samples marked in green.
(C) The right side unidirectional HST with FTD samples marked in green.

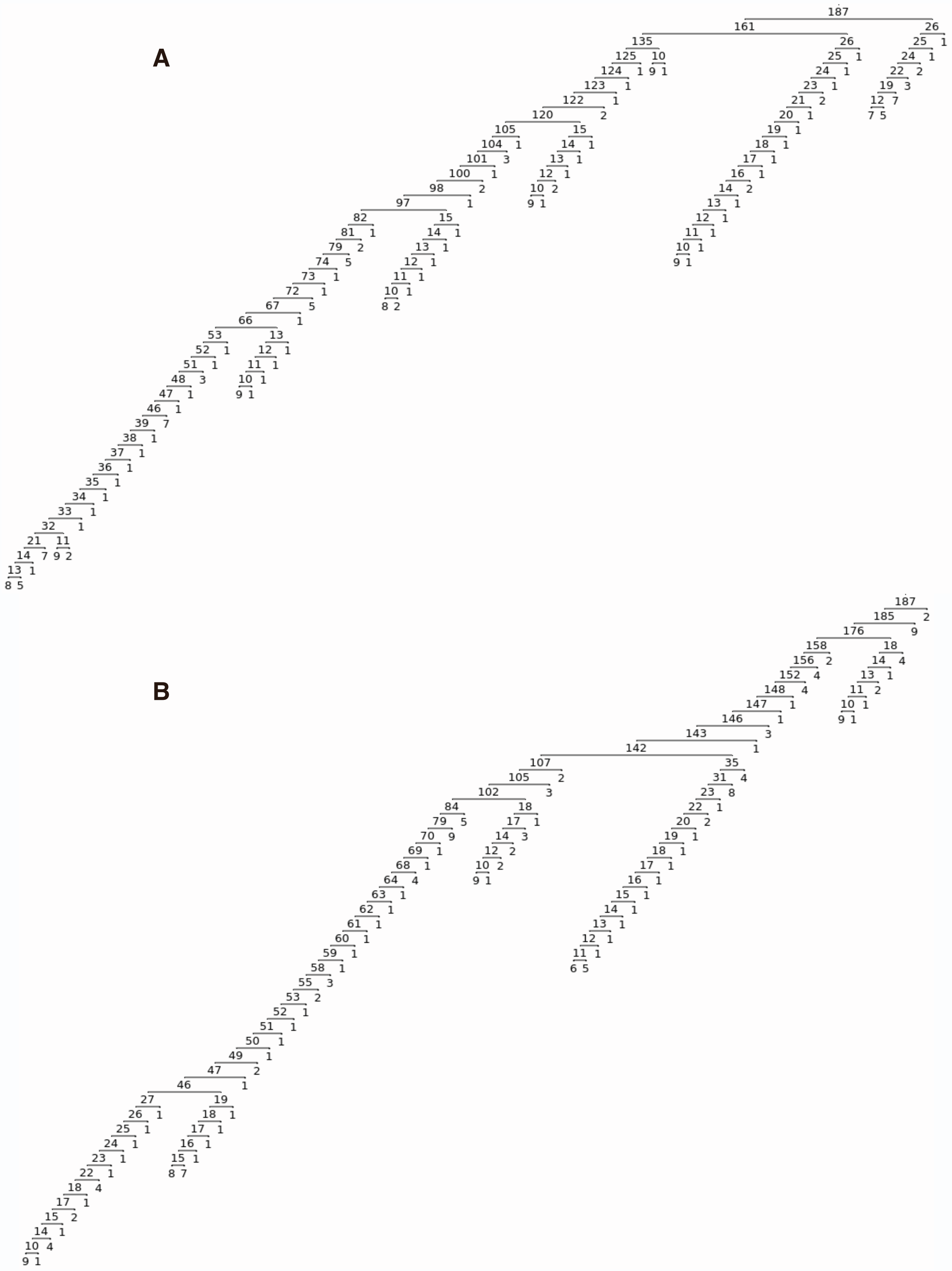

Figure S5. The Helsinki ALS cohort unidirectional HSTs of HRE haplotypes
(A) The right side HST **and (B)** the left side HST **(A and B)** The samples were genotyped with the FinnGen Affymetrix Axiom SNP array. For visualization purposes, the branching is cut after 10 samples.

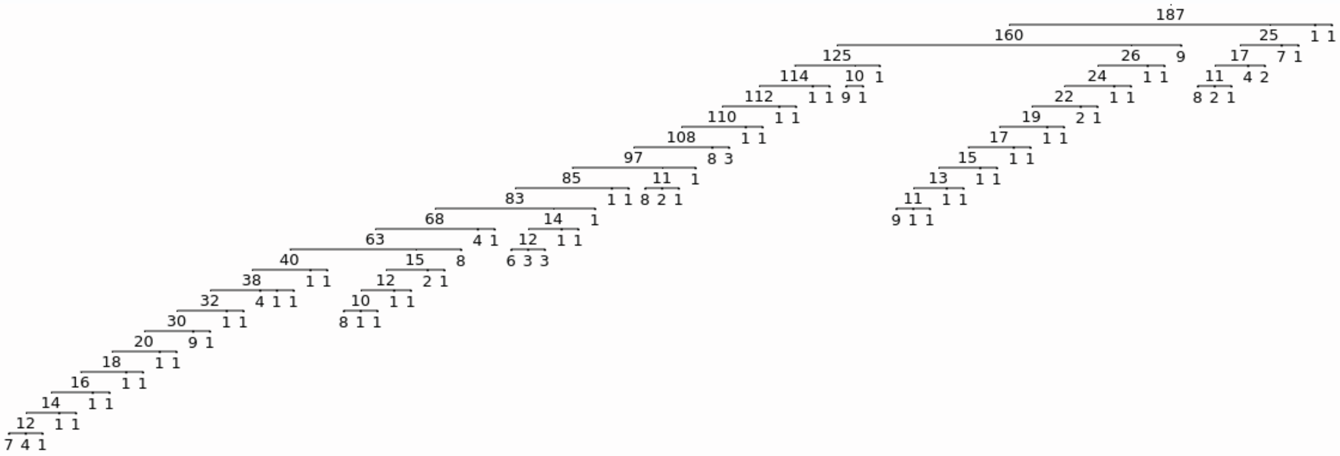

Figure S6. The Helsinki ALS cohort bidirectional HSTs of HRE haplotypes
Genotyped on the custom Affymetrix Axiom SNP array. For visualization purposes, the branching is cut after 10 samples.

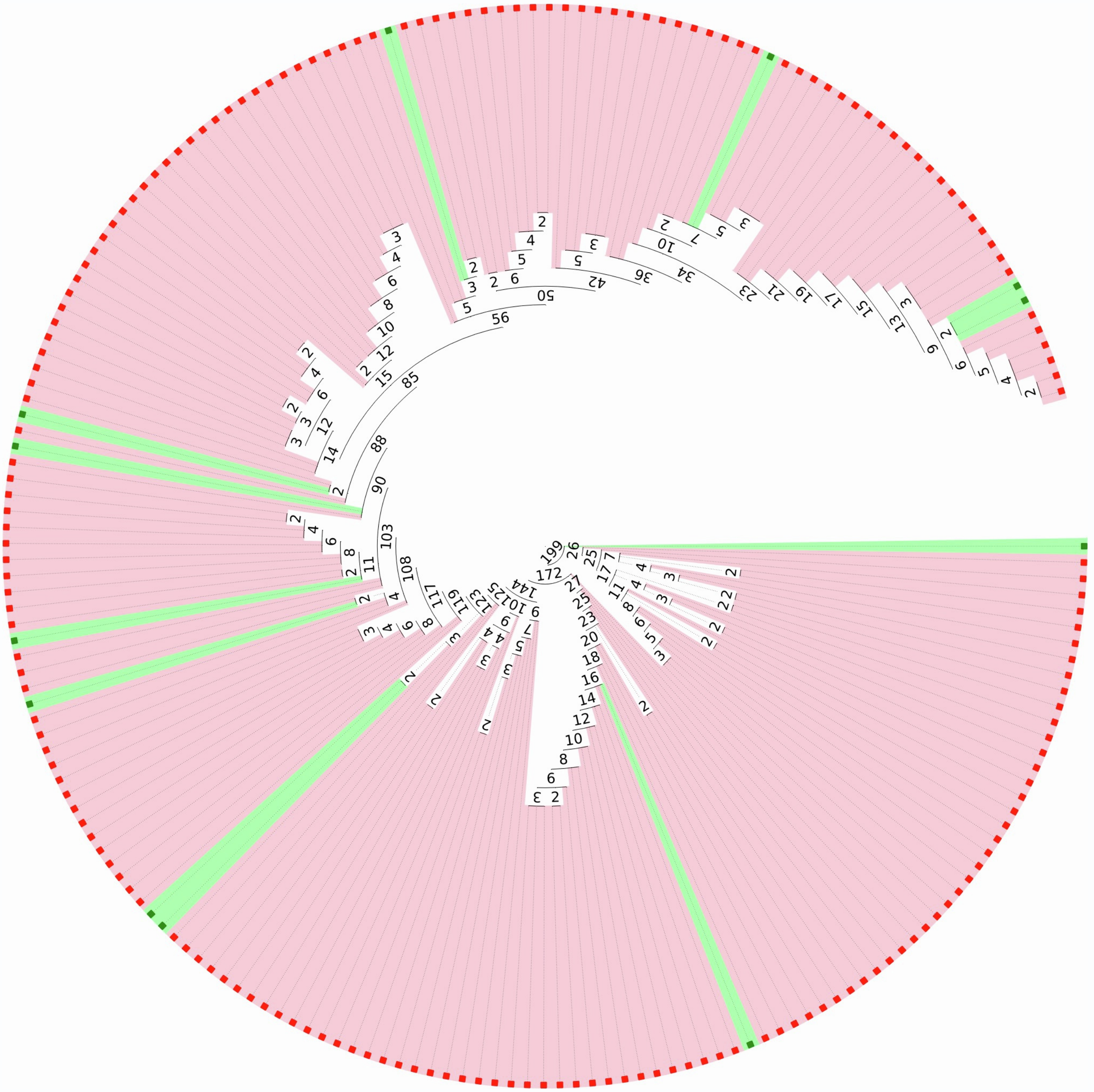

Figure S7. The Helsinki ALS cohort bidirectional HSTs of HREs mixed with ≥20 IAs.
The ≥20 repeat IAs are tagged in green.

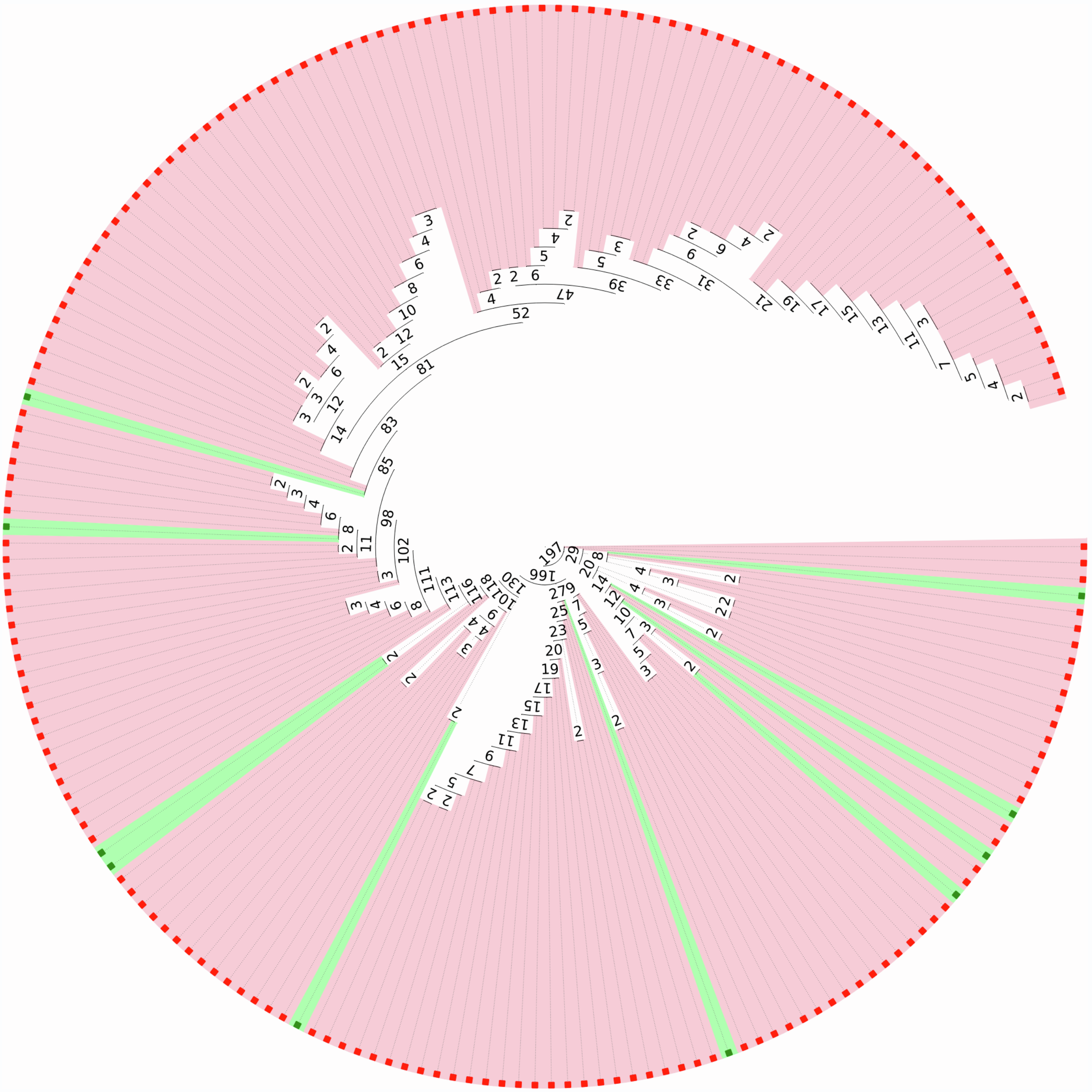

Figure S8. The Helsinki ALS cohort bidirectional HSTs of HREs mixed with 15-19 repeat IAs.
The ≥15 repeat IAs are tagged in green.

Supplemental Tables

Table S1. Genotype concordance between the AmplideX C9orf72 kit and RP-PCR

Table S2. Core HRE haplotype of the Tampere ALS-FTD cohort (Illumina GSA 24v3)

Table S3. Core HRE haplotype of the Helsinki ALS cohort (Affymetrix Axiom custom SNP array)

Table S4. Tampere ALS-FTD ancestral HRE haplotype

Table S5. Helsinki ALS ancestral HRE haplotype

Table S6: Tampere ALS-FTD cohort statistics in groups by GGGGCC repeat size

Table S7. Helsinki ALS cohort statistics in groups by GGGGCC repeat size

Table S8. Tampere ALS-FTD ancestral haplotype compared to Helsinki ALS ancestral haplotype

Table S9. Tampere ALS-FTD ancestral haplotype compared to Helsinki ALS ancestral haplotype with all variants shown

Supplemental Methods

1. Control cohorts

The Helsinki Businessmen study (HBS) included men with high socio-economic status who were born in 1919–1934 and in 2002–2003, 672 individuals who lived at home were randomly selected for analyses (DNA was available in 666).

The DEBATE study was originally a random sample of 4800 individuals from Helsinki out of whom 400 home-living individuals with stable cardiovascular disease were randomly selected for further studies in 2000 (DNA was available for 375). We excluded one individual diagnosed with ALS from these cohorts, which decreased the number of controls from the original 3142 to 3141.

The PLASTICITY cohort is an ongoing long-term follow-up of originally 1196 individuals born in 1971–74 in the Helsinki metropolitan area who had at least one predefined pre- or perinatal risk factor (e.g. low birth weight or Apgar score). 509 subjects were seen at age 40 (DNA available in 433).

2. Unidirectional haplotype sharing tree algorithm

The genotype matrix consists of biallelic phased data without missing genotypes and each sample has two rows of genotypes. The algorithm is run twice, once per direction.

Outline of the algorithm:
1. Select a starting marker from the genotype matrix by a coordinate, if the given coordinate has no designated variant, select the one closest to it.

2. Travel marker by marker towards a given direction from the starting marker.

3. When a contradictory genotype in a column of genotypes is found, assign samples into two new leaf nodes based on the genotypes. For biallelic data, two possible genotypes exist [0], [1].

4. Iterate all leaf nodes.

5. Repeat steps 2. and 3. starting from the last breakpoint and only in the context of the samples present in the currently iterated leaf node.

6. Continue until all leaf nodes have only a single sample left or sequencing data runs out

3. Bidirectional haplotype sharing tree algorithm

The genotype matrix consists of biallelic phased data without missing genotypes and each sample has two rows of genotypes. The algorithm is run once.

Outline of the algorithm:
1. Select a starting marker from the genotype matrix by a coordinate, if the given coordinate has no designated variant, select the one closest to it

2. Travel to the right from the starting marker until a contradictory genotype is found in the column.

3. Travel to the left from the starting marker until a contradictory genotype is found in the column.

4. When both sides present a contradictory genotype, assign samples to four possible genotype buckets: [0,1], [1,0] [0,0] [1,1]. For each bucket, add a new leaf node to the tree.

5. Iterate all leaf nodes.

6. Repeat steps 2. to 4. starting from the last breakpoint and only in the context of the samples present in the currently iterated leaf node.

7. Continue until all leaf nodes have only a single sample left or sequencing data runs out.
