# Supplementary material for: The shared ancestry between the C9orf72 hexanucleotide repeat expansion and intermediate-length alleles using haplotype sharing trees and HAPTK
Source: Am J Hum Genet. 2024 Jan 18;111(2):383–92. doi: 10.1016/j.ajhg.2023.12.019 (PMC10870140; doi:10.1016/j.ajhg.2023.12.019)
Supplement: Document S2. Article plus supplemental information [file mmc3.pdf]

The shared ancestry between the *C9orf72* hexanucleotide repeat expansion and intermediate-length alleles using haplotype sharing trees and HAPTK

Authors

Osma S. Rautila, Karri Kaivola, Harri Rautila, ..., Hannu Laaksovirta, Johanna Palmio, Pentti J. Tienari

Correspondence

osma.rautila@helsinki.fi

Using haplotype sharing trees and majority-based ancestral haplotypes, we demonstrate that the C9orf72 hexanucleotide repeat expansion and alleles with ≥18–20 repeats share large haplotype blocks, indicating very recent shared ancestry. This was in sharp contrast to the shorter alleles, suggesting that expansions might be formed from the longer repeat alleles.

Rautila et al., 2024, The American Journal of Human Genetics *111*, 383–392

February 1, 2024 © 2023 The Authors.

https://doi.org/10.1016/j.ajhg.2023.12.019

CellPress

The shared ancestry between the *C9orf72* hexanucleotide repeat expansion and intermediate-length alleles using haplotype sharing trees and HAPTK

Osma S. Rautila,[1,2,*] Karri Kaivola,[1,2] Harri Rautila,[1] Laura Hokkanen,[4] Jyrki Launes,[4] Timo E. Strandberg,[5] Hannu Laaksovirta,[2] Johanna Palmio,[3] and Pentti J. Tienari[1,2]

Summary

The *C9orf72* hexanucleotide repeat expansion (HRE) is a common genetic cause of amyotrophic lateral sclerosis (ALS) and frontotemporal dementia (FTD). The inheritance is autosomal dominant, but a high proportion of subjects with the mutation are simplex cases. One possible explanation is *de novo* expansions of unstable intermediate-length alleles (IAs). Using haplotype sharing trees (HSTs) with the haplotype analysis tool kit (HAPTK), we derived majority-based ancestral haplotypes of HRE samples and discovered that IAs containing ≥18–20 repeats share large haplotypes in common with the HRE. Using HSTs of HRE and IA samples, we demonstrate that the longer IA haplotypes are largely indistinguishable from HRE haplotypes and that several ≥18–20 IA haplotypes share over 5 Mb (>600 markers) haplotypes in common with the HRE haplotypes. These analysis tools allow physical understanding of the haplotype blocks shared with the majority-based ancestral haplotype. Our results demonstrate that the haplotypes with longer IAs belong to the same pool of haplotypes as the HRE and suggest that longer IAs represent potential premutation alleles.

Introduction

The GGGGCC hexanucleotide repeat expansion (HRE) in the first intron of *C9orf72* (MIM: 614260) is the most common genetic cause of amyotrophic lateral sclerosis (ALS) and frontotemporal dementia (FTD) (FTDALS1 [MIM: 105550]) in populations of European descent.[1–4] Even though the inheritance is autosomal dominant, in many populations, more than half of the individuals with ALS with the *C9orf72* HRE are simplex cases.[3,4] A core haplotype has been reported for the HRE, and the core haplotype, or a tagging SNP, is commonly found in subjects with intermediate-length alleles (IAs, ≥7 repeats), indicating shared ancestry of the HRE and IAs.[4–8] A sequencing-based study from 5 European cohorts defined a core HRE haplotype of 110 kb in ALS and FTD.[5]

The HRE exhibits somatic instability (mosaicism) when repeat lengths have been compared between different tissues.[9] Whether the IAs also show instability is still unclear. However, using the SV40 replication system in transfected cells, DNA replication has been reported to stall at 20 GGGGCC repeats.[10] Stalled DNA replication may recruit alternative, more error-prone replication methods, leading to both contractions and expansions of the repeat sequence.[11] Hence, the longer *C9orf72* IAs could represent unstable premutations as observed in a few other repeat expansion diseases.[12]

If IAs would be unstable premutations generating expansions, then IAs and HRE haplotypes should be identical by descent and belong to the same subfraction of the population. Depending on the speed of the expansion process across generations, the haplotypes with shorter IAs are expected to be genetically more distant from the expanded alleles than haplotypes with longer IAs. We set out to investigate the haplotype sharing between IAs and HRE haplotypes using haplotype sharing trees (HSTs) and majority-based ancestral haplotypes. To streamline the use of HST-based analyses in the future, we developed a haplotype analysis tool kit (HAPTK) written in the programming language Rust using HTSlib bindings from Rust-Bio,[13,14] with tree visualization implemented using ETE 3 and rustworkx.[15,16]

Material and methods

Cohorts and genotyping

To analyze HRE haplotypes, we used the Tampere ALS-FTD cohort (n = 235 HRE subjects, 175 ALS, 55 FTD, and 5 unclear phenotypes) and the Helsinki ALS cohort (n = 202 HRE subjects).[3] For IA genotyping, we selected 1,474 samples from the Finnish population cohort studies HBS, DEBATE, and PLASTICITY[17–19] and 613 non-HRE ALS samples from the Helsinki ALS cohort (see supplemental methods and Figure S1).

The *C9orf72* hexanucleotide repeat lengths were assessed with repeat-primed PCR (RP-PCR) after DNA extraction from peripheral blood leukocytes or saliva (PLASTICITY cohort). The results were visualized using GeneMapper software 6 (ThermoFisher). Because it may be difficult to distinguish between the HRE and a long repeat allele in RP-PCR, we confirmed alleles with ≥20 repeats and all putative expansions with over-the-repeat PCR.[20,21] We used the AmplideX *C9orf72* kit to ascertain a subset of our

[1]Translational Immunology, Research Programs Unit, University of Helsinki, Helsinki, Finland; [2]Department of Neurology, Helsinki University Hospital, Helsinki, Finland; [3]Neuromuscular Research Center, Tampere University and Tampere University Hospital, Tampere, Finland; [4]Department of Psychology and Logopedics, University of Helsinki, Helsinki, Finland; [5]University of Helsinki and Helsinki University Hospital, Helsinki, Finland
*Correspondence: osma.rautila@helsinki.fi
https://doi.org/10.1016/j.ajhg.2023.12.019.
© 2023 The Authors. This is an open access article under the CC BY license (http://creativecommons.org/licenses/by/4.0/).

genotypes and observed high concordance with RP-PCR- and AmplideX-based genotypes (Table S1). The longest non-expanded (amplifiable) discrete allele we could detect was 45 repeats, and we used it as the expansion threshold. Tampere ALS-FTD cohort was genotyped by over-the-repeat PCR and RP-PCR as part of diagnostic testing in the accredited Fimlab laboratories in Tampere.

Illumina Global Screening Array 24v2-3 was used for SNP genotyping for all cohorts except for the Helsinki ALS cohort, which was genotyped with the FinnGen ThermoFisher Axiom custom array. For quality control, we first removed duplicated and related samples (proportion identical by descent [IBD] >0.1875), samples with discordant sex information, outlying heterozygosity rate (>3 standard deviation [SD]), and >5% missing genotype rate. In the per-variant quality control, we included variants that had a genotyping rate of >95%, variants in Hardy-Weinberg equilibrium (HWE) ($p > 0.000001$), and minor allele frequency ≥0.01 or minor allele count >3. To harmonize genotyping array data, we included only biallelic SNPs and set the allele coding to match the GRCh38 reference genome.

After the quality control of each cohort, we merged the cohorts and performed pre-phasing quality control on the merged dataset by excluding markers that had a discrepant allele frequency to the Finnish SISu v3 population reference panel[22] or were not present in the reference panel.[23,24] Reliable HST construction requires the lowest possible switch-error and genotyping error rates. To minimize switch errors, we used Beagle 5.3[25,26] and ran the phasing with 128 iterations with *ne* set to 20,0000. The variants were filtered for missingness (<5%), and the missing genotypes were imputed during phasing. After quality control and duplicate removal, the Tampere ALS-FTD cohort consisted of 142 *C9orf72* HRE ALS, 55 *C9orf72* HRE FTD, and 5 unclear phenotypes, and the Helsinki ALS cohort consisted of 187 individuals with *C9orf72* HRE ALS.

Study approval

This study has been approved by the Institutional Review Boards of the Helsinki University Hospital (Drno 401/13/03/01/2009, HUS/1720/2019). For all cohorts, written informed consent was obtained from each individual or a close relative, depending on whether the individual was physically able to give a written consent. The Tampere ALS-FTD cohort also included diagnostic samples. We have permission from the Finnish Medicines Agency (Drno FIMEA/2020/000180) to analyze diagnostic samples in individuals from whom it was not possible to collect consent in retrospect.

Uni- and bidirectional HSTs

The construction of HSTs requires phased biallelic marker data in the Variant Call Format (VCF). The data is read into a matrix of haplotypes (Figures 1A and 1B). Multiallelic sites should either be removed or split into individual rows in the VCF. In the case of multiallelic loci, the branching is done sequentially depending on which of the multiallelic alleles comes first in the VCF.

Unidirectional HSTs are constructed separately down- and upstream of the starting marker. The algorithm moves along the matrix marker by marker, and if any contradictory genotypes are found in a column of sample genotypes, the samples are branched into two nodes representing the different haplotypes. In contrast, the bidirectional HST is constructed by moving simultaneously in both directions, and when contradictory alleles are found on both sides, the samples are branched into two to four nodes. For both algorithms, the majority of samples are always assigned to the leftmost node. After assigning the new leaf nodes to the branches, the leaf nodes are then recursively processed again to find new contradictory genotypes until all leaf nodes have only a single sample left or the end of genotyping data is reached. The nodes contain information on the branching position and the indexes of the samples sharing the haplotype. Detailed stepwise outlines of the algorithms are found in the supplemental methods.

The HST algorithms only evaluate continuous haplotype sharing, and the HST is a data structure for storing this continuously shared haplotype data. If HSTs are constructed at a locus where an ancestral haplotype is not expected to exist, it must be evaluated whether analyzing continuous haplotype sharing is relevant. It is also of note that the structure of the bidirectional HST is influenced more by the side that has experienced earlier recombinations and that large differences in SNP density on left and right sides of the locus can add some bias to the bidirectional HST.

The majority branch

The leftmost branch of HSTs is called the "majority branch." Following the majority branch, we in a way walk back recombination events in time toward the majority-based ancestral haplotype. The haplotype shared by the last two samples in this branch is the majority-based ancestral haplotype (Figures 1C and 1D). With the unidirectional HSTs, the down- and upstream ancestral haplotypes are merged into a single ancestral haplotype. Here, we refer to the unidirectional HST-derived majority-based ancestral haplotype as the "ancestral haplotype."

Switch error and genotyping error evaluation

The majority-based approach effectively removes switch errors from the ancestral haplotype, as for switch errors to remain, they would need to be in the majority of samples. Therefore, to assess phasing quality, we compare all the haplotypes back against the ancestral haplotype. If long runs of shared ancestral segments are observed after the sample has separated from the majority, a switch error or genotyping error has most likely caused it (Figure S2). To visualize this, we use the ancestral haplotype as a reference sequence and compare all the sample haplotypes to it (Figure 2).

Selecting HRE haplotypes

As no SNPs tag only the HRE,[7] we select the HRE haplotypes by comparing the lengths each phased haplotype shares with the ancestral HRE haplotype. As there is strong evidence of shared ancestry between HRE haplotypes,[4–8] we assume that out of the two possible haplotypes for each sample, the haplotype that shares the longer sequences with the other samples in the cohort of heterozygous HRE haplotypes is the HRE haplotype. Thus, for each sample, we remove the haplotype that shares a shorter segment of the ancestral HRE haplotype (marked in blue in Figure 2). After selecting the haplotype per each sample that shares more sequence in common with the ancestral HRE haplotype, we reconstruct the HST.

MRCA estimation with the gamma method

The lengths of shared segments with the ancestral HRE haplotype can be used to infer how much recombination has occurred in the cohort. This is the basis of the most recent common ancestor (MRCA) estimation with the gamma method.[27] We use the unidirectional HST and register the sharing with the ancestral HRE

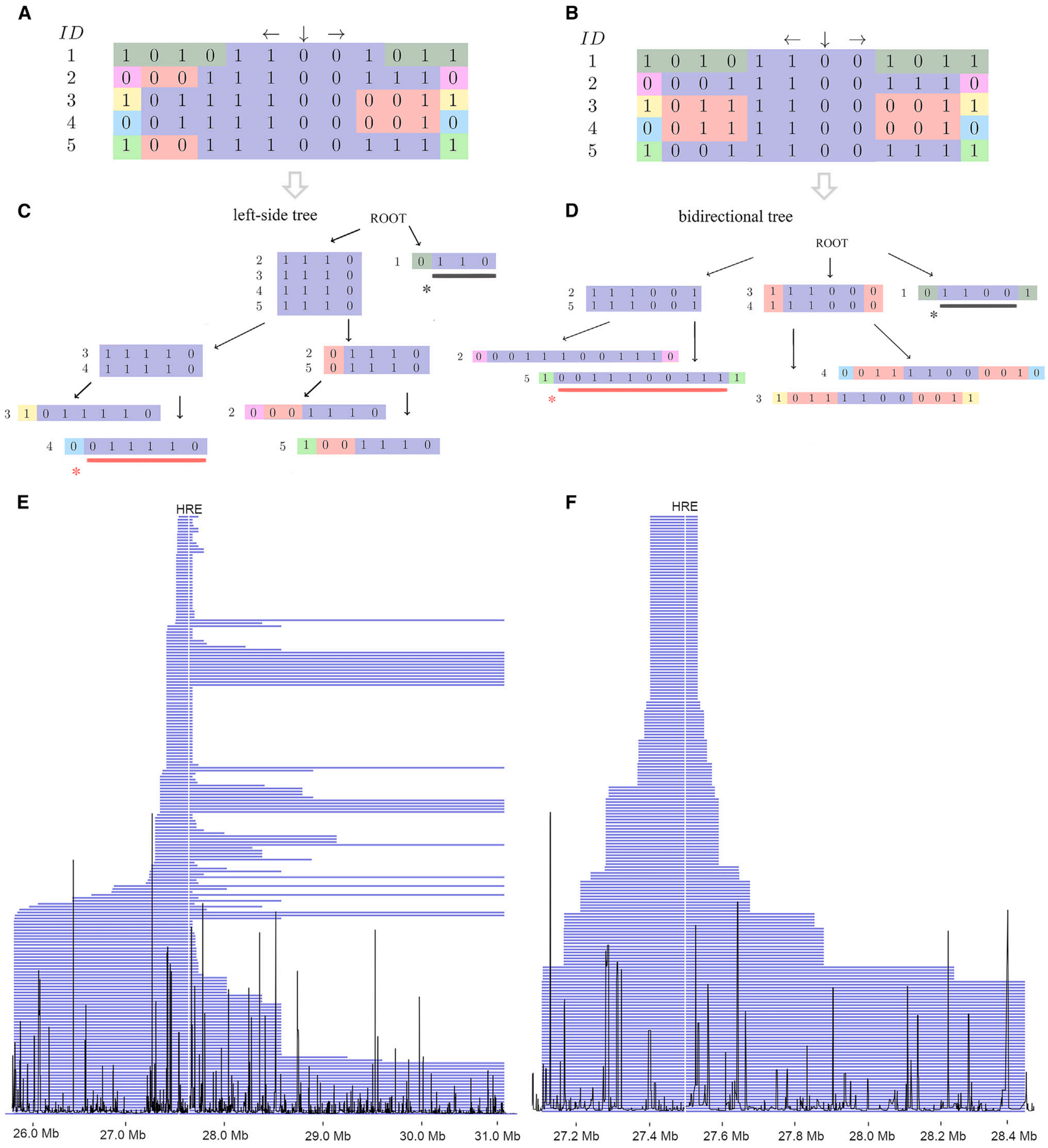

Figure 1. The construction of haplotype sharing trees and Tampere ALS-FTD cohort ancestral haplotype segment lengths per HRE haplotype

(A and B) The genotype matrix with colored first contradictory genotypes from the majority. The matrix is used in the construction of a unidirectional HST (A) and (B) the bidirectional HST. Each column of the matrix is a biallelic SNP marker and each row represents a phased haplotype. The down arrow denotes the starting marker of the algorithm.

(C and D) Illustration of the construction of the left-side unidirectional HST (C) and (D) the bidirectional HST. The construction of the right-side tree is not shown here.* The core haplotype is underlined in black, and the majority-based ancestral haplotype is underlined in red. When using unidirectional HSTs, the ancestral and core haplotypes from both sides need to be combined into a single core and a single ancestral haplotype.

(E and F) The unidirectional HST-derived ancestral haplotype segment lengths (E) and the (F) bidirectional HST-derived ancestral haplotype segment lengths. Segment lengths of the ancestral haplotype for each sample after selecting only the longer ancestral sequence sharing haplotypes. Recombination frequencies are drawn in black and seem to correspond to the sites of haplotype breaks. The vertical white line marks the HRE locus. For visualization purposes, the x axis is limited to positions where at least 40 samples are sharing the haplotype.

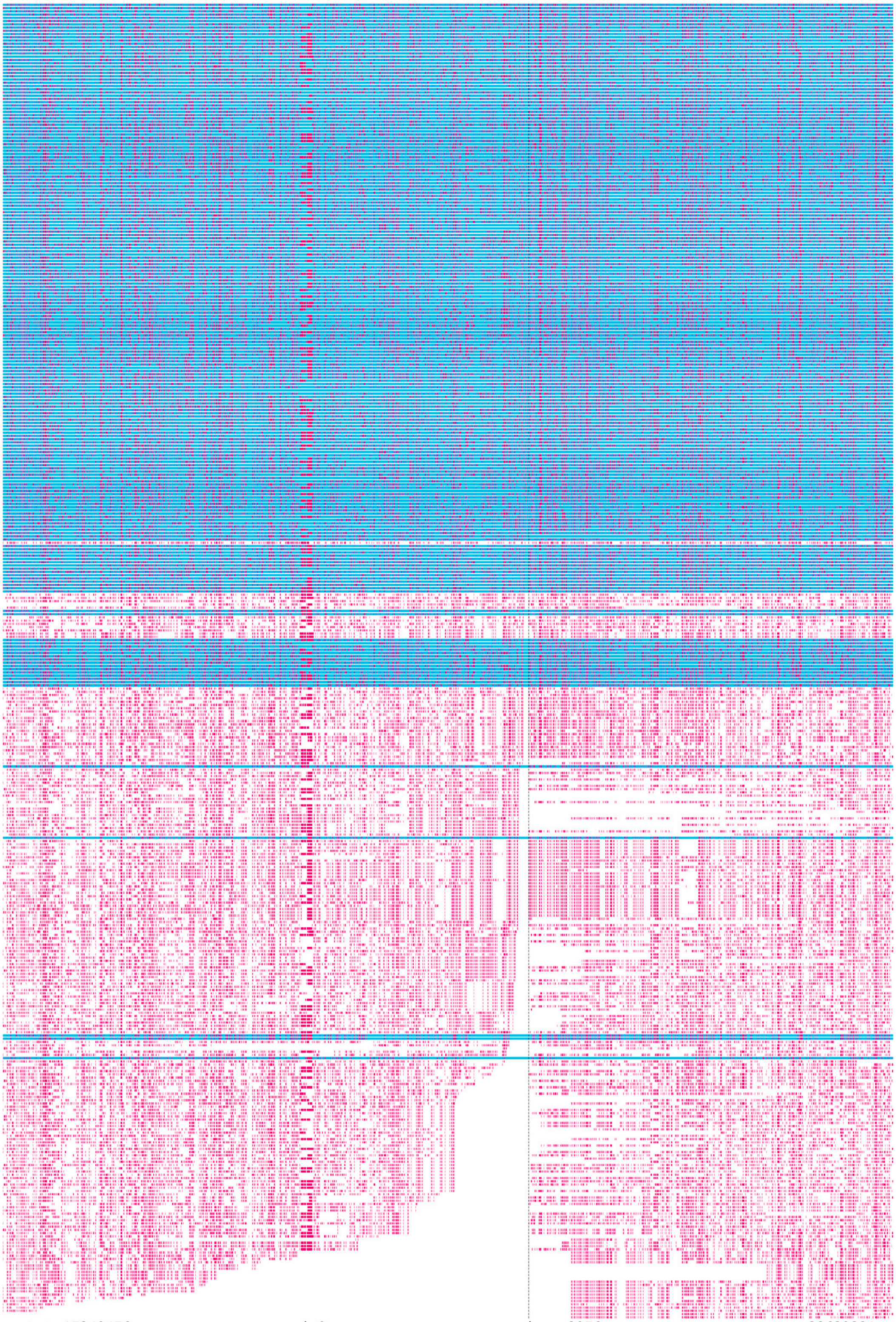

Figure 2. The comparison of all Tampere ALS-FTD cohort haplotypes to the unidirectional HST-derived ancestral haplotype
All phased haplotypes of the Tampere ALS-FTD cohort are compared to the majority-based ancestral haplotype as the reference. Alternative alleles are colored in pink. The reference alleles are colored in blue or white. We use blue for the haplotype sharing a shorter segment of the ancestral haplotype per sample and white for the one sharing a longer segment. The selection of HRE haplotypes is done by discarding the haplotypes marked in blue. As *C9orf72* HRE inheritance is autosomal dominant and no HRE homozygotes are present, theoretically, all the blue haplotypes should be on the top. However, some IA haplotypes share longer segments of the ancestral HRE haplotype than other HRE haplotypes. For example, the lowest three haplotypes marked in blue have both a $\geq$20 repeat IA and the HRE.

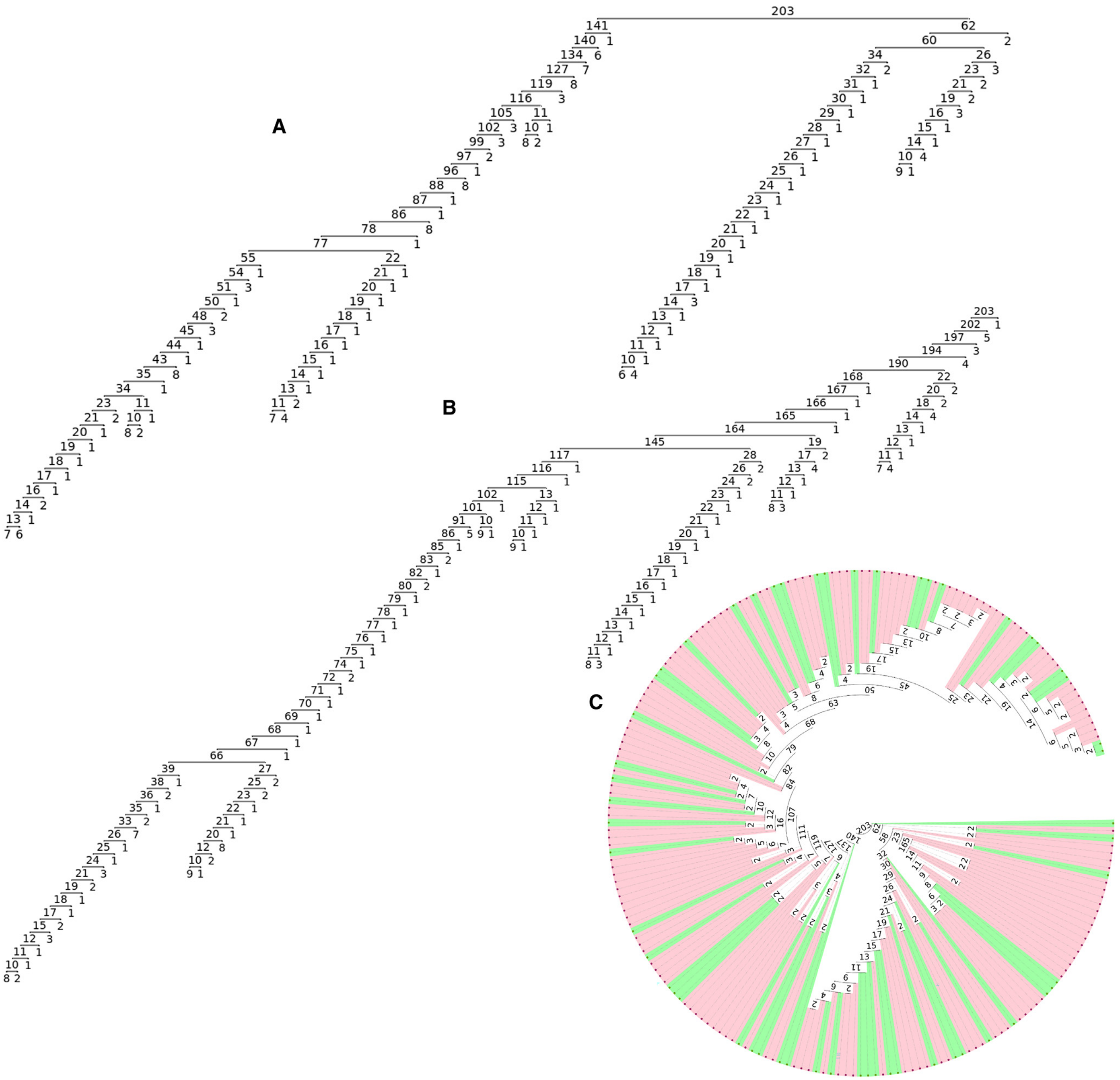

Figure 3. Tampere ALS-FTD cohort HSTs of HRE haplotypes
(A and B) The numbers denote the number of samples sharing the haplotype of the node. For visualization purposes, the branching is stopped for the unidirectional HSTs after nodes have fewer than 10 remaining samples. (A) The right side (upstream) unidirectional HST. The first marker breaking the core haplotype divides the samples to two large haplotype subtrees (141 vs. 62). (B) The left side (downstream) unidirectional HST. Four to five smaller subtrees are formed from the majority.
(C) The bidirectional HST with FTD samples tagged in green. The FTD subjects are quite uniformly distributed along the tree and do not form any clear subtrees.

haplotype for each sample. We then translate the distance between the breakpoint from the ancestral haplotype and the HRE locus to centimorgans using Beagle genetic maps. To estimate the MRCA, we ported the original algorithm without chance-sharing correction from R into our application.

Analysis of the HRE and IA ancestries

The IAs were categorized into predefined groups of 2–6, 7–9, 10–14, 15–19, and 20–45 repeats.[7] The cutoff point of 7 repeats is commonly used to define the smallest IA, the cutoff 10 is based on a peak in allele frequency, the cutoff 15 is based on a drop in allele frequency, and the cutoff 20 is based on a tagging SNP.[7] The samples were grouped based on the largest repeat. To estimate a possible threshold effect, we made an additional analysis with discrete IAs.

Cohort-to-haplotype and cohort-to-HST comparisons

After removing all identifications and haplotypes shared between fewer than 10 individuals, we published the Tampere FTD-ALS cohort HSTs to a freely accessible repository.[28] We then analyzed

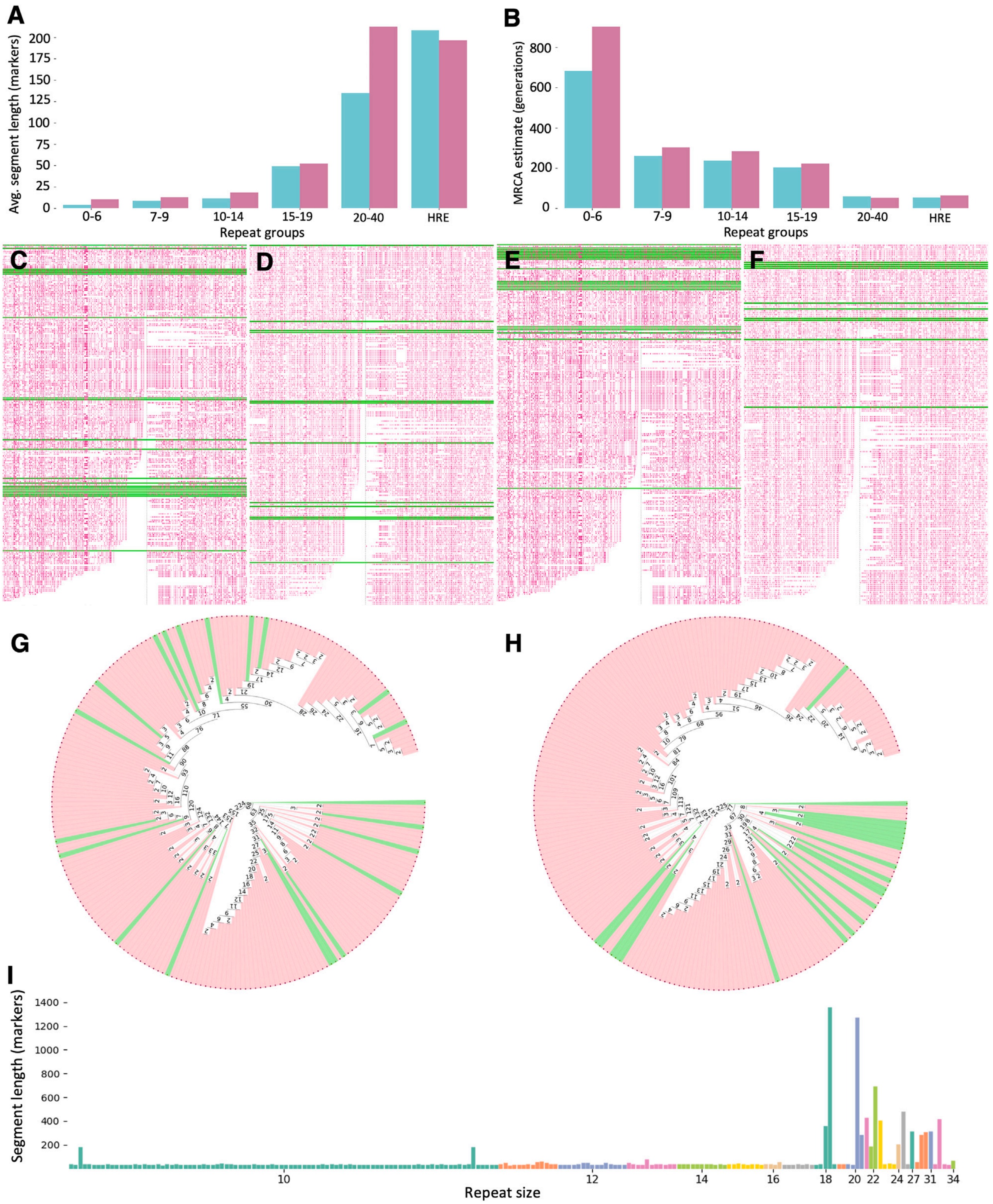

Figure 4. Ancestral HRE haplotype sharing in the IA groups

(A and B) The Tampere FTD-ALS cohort in cyan and the Helsinki ALS cohort in purple. (A) Average ancestral HRE haplotype segment length in markers per group. The groups include all haplotypes, not only the longer ancestral segment sharing ones. In the Helsinki ALS cohort in the ≥20 IA group, nine out of twelve had two ≥7 IAs, raising the average segment sharing over the HRE group itself. (B) MRCA estimates of the longer ancestral sequence sharing haplotypes per group in generations using the gamma method with correlated genealogy.

(C–F) HRE haplotypes are mixed with IA haplotypes and compared against the ancestral HRE haplotype. IA haplotypes are marked in green. (C) Tampere ALS-FTD cohort HRE haplotypes mixed with ≥20 repeat IAs. (D) Helsinki ALS cohort HRE haplotypes mixed

(legend continued on next page)

the haplotype sharing of the Helsinki ALS cohort haplotypes with the Tampere ALS-FTD cohort ancestral haplotype and tested how the HRE haplotypes of the Helsinki ALS cohort compared to the Tampere ALS-FTD bidirectional HST of HRE haplotypes.

Results

We constructed the HSTs and derived the core and ancestral HRE haplotypes using a starting base pair position 27,582,313 (GRCh38), which was the closest marker upstream of the *C9orf72* HRE. In the Tampere ALS-FTD cohort, we found a 13-SNP 114-kb core haplotype shared between all HRE haplotypes (Table S2). In the Helsinki ALS cohort, we found a 10-SNP 89-kb core HRE haplotype (Table S3, a different SNP array was used). We visualized the Tampere ALS-FTD cohort ancestral HRE haplotype lengths for each individual sample based on the haplotype length at the last node before diverging from the majority branch in Figures 1E and 1F.

To assess the quality of the phasing and the integrity of the HSTs, we compared all the haplotypes against the unidirectional HST-derived ancestral haplotype (Table S4). The comparison graph was sorted by the amount of downstream (left side) haplotype sharing between each haplotype and the ancestral HRE haplotype (Figure 2). Based on the comparison graph, switch-error rate was visually determined to be low, and we chose not to try to correct the few potential switch errors remaining in the data. The suspected switch errors are pointed out in Figure S2. The selection of HRE haplotypes based on ancestral HRE haplotype sharing was deemed adequate (Figure 2).

Next, we visualized the unidirectional (Figures 3A, 3B, S5A, and S5B) and bidirectional (Figures S3 and S6) HSTs. Our data indicated a relatively early upstream recombination, and the Tampere ALS-FTD cohort seemed to form two subtrees (Figure 3). Expression of the HRE allele is variable and depends on its methylation status.[29,30] Hence, we tested whether the haplotype background of the HRE would differ in FTD and ALS by marking the individuals with FTD in the uni- and bidirectional HSTs. However, the FTD-related haplotypes did not form any clear subtrees, indicating that the haplotype composition is not a major determinant of whether an individual develops ALS or FTD (Figures 3C and S4).

We then compared the predefined groups of IAs to the ancestral HRE haplotype to analyze whether the IA groups would differ in haplotype sharing with the HRE. We found most haplotype sharing in the ≥ 20 repeat IA category in both cohorts. Haplotype sharing strongly diminished already in the 15–19 repeat IA category and continued to decrease toward the 2–6 repeat category (Figure 4A; Tables S6 and S7). To detect a possible threshold effect, we analyzed the ancestral HRE haplotype sharing for all haplotypes with ≥ 10 repeat discrete IAs. A threshold effect was seen in IA haplotypes starting at 18 repeats (Figure 4I).

Next, we compared all the haplotypes to the ancestral HRE haplotype (Table S4) and marked the IAs in green. The ≥ 20 repeat IAs distributed relatively evenly along the graph in both cohorts (Figures 4C and 4D). On the other hand, most of the 15–19 repeat IAs diverged from the ancestral HRE haplotype quite early (Figures 4E and 4F). To detect possible division into subtrees, we constructed a bidirectional HST of the ≥ 20 repeat IAs mixed with HRE alleles and marked the IA haplotypes in green. We found that the ≥ 20 repeat IAs were spread across the HSTs and did not form any clear subtrees (Figures 4G and S7), whereas most of the 15–19 repeat alleles diverged relatively early from the majority branch (Figures 4H and S8).

Then, we estimated the MRCA with the gamma method under correlated genealogy and found that in the shorter groups the MRCA was high, but in both cohorts in the ≥ 20 IA group, it was close to the HRE group (Figure 4B). The MRCAs of the HRE haplotypes were ca. 54.6 (9.7–98.1, 95% confidence interval [CI], Tampere ALS-FTD cohort) and ca. 65.3 (14.8–114.7, 95% CI, Helsinki ALS cohort) generations ago. Assuming 25 years per generation, these figures correspond to 1,365 years (243–2,453 years, 95% CI) in the Tampere ALS-FTD cohort and 1,633 years (370–2,868 years, 95% CI) in the Helsinki ALS cohort.

Finally, we compared the Helsinki ALS cohort HRE haplotypes to the Tampere ALS-FTD cohort HST we priorly published.[28] The Helsinki dataset had 373 markers in common with the Tampere ALS-FTD ancestral HRE haplotype. A visualization of the shared haplotype lengths is shown in Figures 5A and 5B. The mean segment sharing between the Helsinki ALS cohort haplotypes with the Tampere ancestral HRE haplotype was 2.47 megabases (Mb) (Figure 5C). The Helsinki haplotypes distributed along the Tampere HST in similar proportions as the Tampere haplotypes and all the Tampere HST haplotypes were also found in the Helsinki cohort (Figures 5D and 5E).

Discussion

When comparing the ancestral HRE haplotype sharing in different length IAs, we found significantly increased haplotype sharing starting from 18 to 20 repeats. These findings demonstrate more recent shared ancestry between the longer IAs and the HRE. More than half of the individuals with ALS with the *C9orf72* HRE are simplex

with ≥ 20 repeat IAs. (E) Tampere ALS-FTD cohort HRE haplotypes mixed with 15–19 repeat IAs. (F) Helsinki ALS cohort HRE haplotypes mixed with 15–19 repeat IAs.
(G) A bidirectional HST of Tampere ALS-FTD cohort HRE haplotypes mixed with ≥ 20 IAs (marked in green).
(H) As in (G), but HRE haplotypes mixed with 15–19 IAs.
(I) Ancestral HRE haplotype segment lengths (y axis) in ≥ 10 repeat groups for both phased chromosomes of all the Tampere ALS-FTD and population control samples. For visualization purposes, all haplotypes without the core HRE haplotype were filtered out.

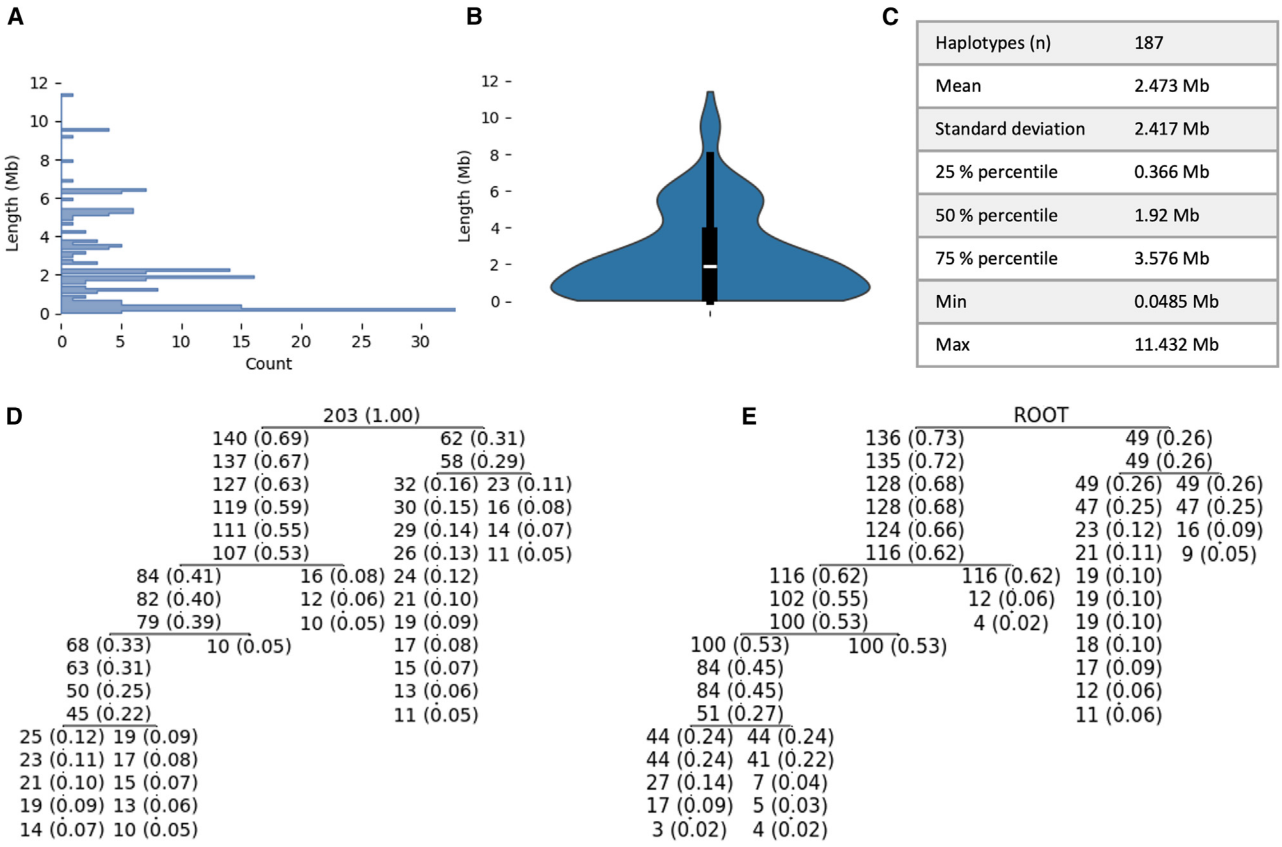

Figure 5. Cohort-to-haplotype and cohort-to-HST comparisons

(A) Haplotype sharing (in megabases) between the Tampere ALS-FTD ancestral HRE haplotype and the Helsinki ALS cohort HRE haplotypes.

(B) (A) represented as a violin plot.

(C) Summary statistics of the haplotype sharing lengths in relation to the Tampere ALS-FTD ancestral HRE haplotype. It is of note that the arrays only shared 373 markers in common of the Tampere ALS-FTD ancestral HRE haplotype.

(D) Tampere ALS-FTD cohort bidirectional HST with haplotypes shared by at least 10 samples (available in Zenodo). The proportion of samples is in parentheses.

(E) Comparison of the 187 samples of the Helsinki ALS cohort to the Tampere ALS-FTD cohort bidirectional HST. First, we selected only the HRE haplotypes from the Helsinki ALS cohort. Then, for each haplotype (i.e., node) in the Tampere ALS-FTD bidirectional HST, we analyzed how many of the Helsinki ALS cohort HRE haplotypes share that haplotype. Note that as shown in Figures 1C and 1D, the root node has no haplotype, so it cannot be compared against. If two branches have an equal number of samples at the same level, then the markers differentiating the haplotypes in the Tampere ALS-FTD cohort were not present in the Helsinki ALS cohort dataset.

cases,[3,4] and one hypothesis for this observation is a parental premutation, i.e., an unstable IA.[6,7,20,31] Previously, one family has been described in which a ~70-repeat allele from the unaffected father expanded during parent-offspring transmission and started the first generation affected by ALS.[31] Our data indicate that even smaller and more common repeat alleles may represent unstable premutations. The population frequency of $\geq$20 repeat alleles is around 1.7%,[21] and the frequency of the HRE in older population is in the range of 0.2%.[20] Given the high degree of haplotype sharing between HRE and longer IAs, our data support the concept that the expanded alleles are formed from the longer IAs. However, we cannot rule out whether some IAs are formed from contracted expansions. These findings align with studies showing that at 20 GGGGCC repeats, the DNA replication system becomes more error prone.[10]

This study has certain limitations. First, some IA alleles share long ancestral HRE haplotypes, and as some HRE samples also have an IA, the selection of the longer ancestral HRE haplotype segment does not mean it is certain that the HRE allele is selected. For example, we found three subjects with both the HRE and $\geq$20-repeat IAs, but when tested, the removal of these samples did not affect the ancestral haplotype. Second, a few possible switch errors were seen in the comparison graphs. When the samples with the suspected switch errors were removed, the majority-based ancestral HRE haplotype was not affected. Also, as there are no gross switch errors in IA groups and the switch-error rate is expected to be relatively uniform across all repeat groups, we did not try to correct for these possible switch errors.

Regarding ancestral haplotype sharing, the uni- and bidirectional HST-derived analyses give similar results.

The bidirectional approach may not be suitable for MRCA estimation, and when analyzing the effects of these shared haplotypes on phenotypic traits, we find it good practice to also evaluate the unidirectional HSTs. Unidirectional HSTs may be better for evaluating possible biological effects when variation either only up- or downstream of the starting site is expected to drive the phenotype. When only the general amount of haplotype sharing is analyzed or a set of shared haplotypes needs to be quickly constructed, we prefer the bidirectional approach for the simplicity of a single tree. The future use for bidirectional HSTs includes haplotype block-based genome-wide association studies by stepwise genome-wide construction of HSTs and then by minimizing each tree for a minimum node value (in associations studies, the p value from comparing node samples to the rest of the samples).

In the future, we still aim to make several improvements to the HST standard and HAPTK functionality. Although the analyses on these cohort sizes were run within minutes on consumer-grade hardware and memory usage was negligible, several optimizations remain to be done to allow for efficient use with biobank scale data.

We have published the fully anonymized Tampere ALS-FTD HSTs (all identification and sample indexing information removed) and made them freely available.[28] For additional personal data protection rights reasons, we have chosen to only include haplotypes shared by at least 10 individuals. We hope that HSTs from different loci of interest, related to all types of inherited disease, would in the future be publicly available in the HST—or any other—format to ease the analysis of haplotype sharing between different cohorts globally.

Data and code availability

The Tampere ALS-FTD cohort HSTs are available in Zenodo (https://doi.org/10.5281/zenodo.10060939).

The source code for HAPTK and all the scripts used in this article are available at https://github.com/xosxos/haptk.

Supplemental information

Supplemental information can be found online at https://doi.org/10.1016/j.ajhg.2023.12.019.

Acknowledgments

We thank all the study participants for their generous participation in the ALS, PLASTICITY, DEBATE, and HBS studies. The data used for the research was phased with the THL Biobank's SISu v3 Imputation reference panel obtained from THL Biobank. We thank the Sequencing Informatics Team, FIMM Human Genomics, University of Helsinki, and the participants of the FINRISK, Health 2000, and Migraine Family studies for the work done in preparation of the reference panel data. Thank you to Dr. Joona Sarkkinen, Dr. Jani Huuhtanen, and Dr. Sofie Lundgren for information on Zenodo and other data sharing-related inquiries.

This study was funded by the Finnish Cultural Foundation, Päivikki and Sakari Sohlberg Foundation, Paulo Foundation, the Sigrid Jusélius Foundation, Maire Taponen Foundation, the Finnish Brain Foundation, the Finnish Medical Foundation, the Biomedicum Helsinki Foundation, Helsinki University Hospital grants, ALS tuttu ry and the Finnish Academy (318868).

Declaration of interests

P.J.T. holds a patent on C9orf72 in diagnostics and treatment of ALS/FTD.

Received: September 8, 2023
Accepted: December 18, 2023
Published: January 18, 2024

References

1. DeJesus-Hernandez, M., Mackenzie, I.R., Boeve, B.F., Boxer, A.L., Baker, M., Rutherford, N.J., Nicholson, A.M., Finch, N.A., Flynn, H., Adamson, J., et al. (2011). Expanded GGGGCC hexanucleotide repeat in noncoding region of C9ORF72 causes chromosome 9p-linked FTD and ALS. Neuron *72*, 245–256.
2. Renton, A.E., Majounie, E., Waite, A., Simón-Sánchez, J., Rollinson, S., Gibbs, J.R., Schymick, J.C., Laaksovirta, H., Van Swieten, J.C., Myllykangas, L., et al. (2011). A hexanucleotide repeat expansion in C9ORF72 is the cause of chromosome 9p21-linked ALS-FTD. Neuron *72*, 257–268.
3. Laaksovirta, H., Launes, J., Jansson, L., Traynor, B.J., Kaivola, K., and Tienari, P.J. (2022). ALS in Finland: Major Genetic Variants and Clinical Characteristics of Patients With and Without the C9orf72 Hexanucleotide Repeat Expansion. Neurol. Genet. *8*, e665.
4. Majounie, E., Renton, A.E., Mok, K., Dopper, E.G.P., Waite, A., Rollinson, S., Chiò, A., Restagno, G., Nicolaou, N., Simon-Sanchez, J., et al. (2012). Frequency of the C9orf72 hexanucleotide repeat expansion in patients with amyotrophic lateral sclerosis and frontotemporal dementia: a cross-sectional study. Lancet Neurol. *11*, 323–330.
5. Smith, B.N., Newhouse, S., Shatunov, A., Vance, C., Topp, S., Johnson, L., Miller, J., Lee, Y., Troakes, C., Scott, K.M., et al. (2013). The C9ORF72 expansion mutation is a common cause of ALS+/- FTD in Europe and has a single founder. Eur. J. Hum. Genet. *21*, 102–108.
6. Reus, L.M., Jansen, I.E., Mol, M.O., van Ruissen, F., Holstege, H., van Rooij, J., van Schoor, N.M., Tesi, N., Reinders, M.J.T., et al. (2021). Genome-wide association study of frontotemporal dementia identifies a C9ORF72 haplotype with a median of 12-G4C2 repeats that predisposes to pathological repeat expansions. Transl. Psychiatry *11*, 451.
7. Kaivola, K., Pirinen, M., Laaksovirta, H., Jansson, L., Rautila, O., Launes, J., Hokkanen, L., Lahti, J., Eriksson, J.G., Strandberg, T.E., et al. (2023). C9orf72 hexanucleotide repeat allele tagging SNPs: Associations with ALS risk and longevity. Front. Genet. *14*, 1087098.
8. van der Zee, J., Gijselinck, I., Dillen, L., Van Langenhove, T., Theuns, J., Engelborghs, S., Philtjens, S., Vandenbulcke, M., Sleegers, K., Sieben, A., et al. (2013). A Pan-European Study of the C9orf72 Repeat Associated with FTLD: Geographic

Prevalence, Genomic Instability, and Intermediate Repeats. Hum. Mutat. *34*, 363–373.
9. Fratta, P., Polke, J.M., Newcombe, J., Mizielinska, S., Lashley, T., Poulter, M., Beck, J., Preza, E., Devoy, A., Sidle, K., et al. (2015). Screening a UK amyotrophic lateral sclerosis cohort provides evidence of multiple origins of the C9orf72 expansion. Neurobiol. Aging *36*, 546.e1–546.e7.
10. Thys, R.G., and Wang, Y.-H. (2015). DNA replication dynamics of the GGGGCC repeat of the C9orf72 gene. J. Biol. Chem. *290*, 28953–28962.
11. Mirkin, E.V., and Mirkin, S.M. (2007). Replication fork stalling at natural impediments. Microbiol. Mol. Biol. Rev. *71*, 13–35.
12. Pearson, C.E., Nichol Edamura, K., and Cleary, J.D. (2005). Repeat instability: mechanisms of dynamic mutations. Nat. Rev. Genet. *6*, 729–742.
13. Bonfield, J.K., Marshall, J., Danecek, P., Li, H., Ohan, V., Whitwham, A., Keane, T., and Davies, R.M. (2021). HTSlib: C library for reading/writing high-throughput sequencing data. GigaScience *10*.
14. Köster, J. (2016). Rust-Bio: a fast and safe bioinformatics library. Bioinformatics *32*, 444–446.
15. Huerta-Cepas, J., Serra, F., and Bork, P. (2016). ETE 3: Reconstruction, Analysis, and Visualization of Phylogenomic Data. Mol. Biol. Evol. *33*, 1635–1638.
16. Treinish, M., Carvalho, I., Tsilimigkounakis, G., and Sá, N. (2022). rustworkx: A High-Performance Graph Library for Python. J. Open Source Softw. *7*, 3968.
17. Strandberg, T.E., Salomaa, V., Strandberg, A.Y., Vanhanen, H., Sarna, S., Pitkälä, K., Rantanen, K., Savela, S., Pienimäki, T., Huohvanainen, E., et al. (2016). Cohort profile: the Helsinki businessmen study (HBS). Int. J. Epidemiol. *45*, 1074. 1074h.
18. Hokkanen, L., Launes, J., and Michelsson, K. (2013). The Perinatal Adverse events and Special Trends in Cognitive Trajectory (PLASTICITY)-pre-protocol for a prospective longitudinal follow-up cohort study. F1000Res. *2*, 50.
19. Niemi, A.-K., Hervonen, A., Hurme, M., Karhunen, P.J., Jylhä, M., and Majamaa, K. (2003). Mitochondrial DNA polymorphisms associated with longevity in a Finnish population. Hum. Genet. *112*, 29–33.
20. Kaivola, K., Kiviharju, A., Jansson, L., Rantalainen, V., Eriksson, J.G., Strandberg, T.E., Laaksovirta, H., Renton, A.E., Traynor, B.J., Myllykangas, L., and Tienari, P.J. (2019). C9orf72 hexanucleotide repeat length in older population: normal variation and effects on cognition. Neurobiol. Aging *84*, 242.e7. 242242.e12.
21. Kaivola, K., Salmi, S.J., Jansson, L., Launes, J., Hokkanen, L., Niemi, A.-K., Majamaa, K., Lahti, J., Eriksson, J.G., Strandberg, T., et al. (2020). Carriership of two copies of C9orf72 hexanucleotide repeat intermediate-length alleles is a risk factor for ALS in the Finnish population. Acta Neuropathol. Commun. *8*, 187–189.
22. Lim, E.T., Würtz, P., Havulinna, A.S., Palta, P., Tukiainen, T., Rehnström, K., Esko, T., Mägi, R., Inouye, M., Lappalainen, T., et al. (2014). Distribution and medical impact of loss-of-function variants in the Finnish founder population. PLoS Genet. *10*, e1004494.
23. Zuvich, R.L., Armstrong, L.L., Bielinski, S.J., Bradford, Y., Carlson, C.S., Crawford, D.C., Crenshaw, A.T., de Andrade, M., Doheny, K.F., Haines, J.L., et al. (2011). Pitfalls of merging GWAS data: lessons learned in the eMERGE network and quality control procedures to maintain high data quality. Genet. Epidemiol. *35*, 887–898.
24. Pärn, K., Isokallio, M. A., Fontarnau, J. N., Palotie, A., Ripatti, S., and Palta, P. Genotype Imputation Workflow v3.0 V.2. 10.17504/protocols.io.xbgfijw
25. Oget-Ebrad, C., Kadri, N.K., Moreira, G.C.M., Karim, L., Coppieters, W., Georges, M., and Druet, T. (2022). Benchmarking phasing software with a whole-genome sequenced cattle pedigree. BMC Genom. *23*, 130–216.
26. Browning, B.L., Tian, X., Zhou, Y., and Browning, S.R. (2021). Fast two-stage phasing of large-scale sequence data. Am. J. Hum. Genet. *108*, 1880–1890.
27. Gandolfo, L.C., Bahlo, M., and Speed, T.P. (2014). Dating rare mutations from small samples with dense marker data. Genetics *197*, 1315–1327.
28. Rautila, O.S., Kaivola, K., Rautila, H., Hokkanen, L., Strandberg, T.E., Launes, J., Laaksovirta, H., Palmio, J., and Tienari, P. (2023). Tampere ALS-FTD Cohort HSTs.
29. Liu, E.Y., Russ, J., Wu, K., Neal, D., Suh, E., McNally, A.G., Irwin, D.J., Van Deerlin, V.M., and Lee, E.B. (2014). C9orf72 hypermethylation protects against repeat expansion-associated pathology in ALS/FTD. Acta Neuropathol. *128*, 525–541.
30. Xi, Z., Zinman, L., Moreno, D., Schymick, J., Liang, Y., Sato, C., Zheng, Y., Ghani, M., Dib, S., Keith, J., et al. (2013). Hypermethylation of the CpG island near the G4C2 repeat in ALS with a C9orf72 expansion. Am. J. Hum. Genet. *92*, 981–989.
31. Xi, Z., van Blitterswijk, M., Zhang, M., McGoldrick, P., McLean, J.R., Yunusova, Y., Knock, E., Moreno, D., Sato, C., McKeever, P.M., et al. (2015). Jump from pre-mutation to pathologic expansion in C9orf72. Am. J. Hum. Genet. *96*, 962–970.

The American Journal of Human Genetics, Volume *111*

Supplemental information

The shared ancestry between the *C9orf72* hexanucleotide repeat expansion and intermediate-length alleles using haplotype sharing trees and HAPTK

Osma S. Rautila, Karri Kaivola, Harri Rautila, Laura Hokkanen, Jyrki Launes, Timo E. Strandberg, Hannu Laaksovirta, Johanna Palmio, and Pentti J. Tienari

Supplemental Figures

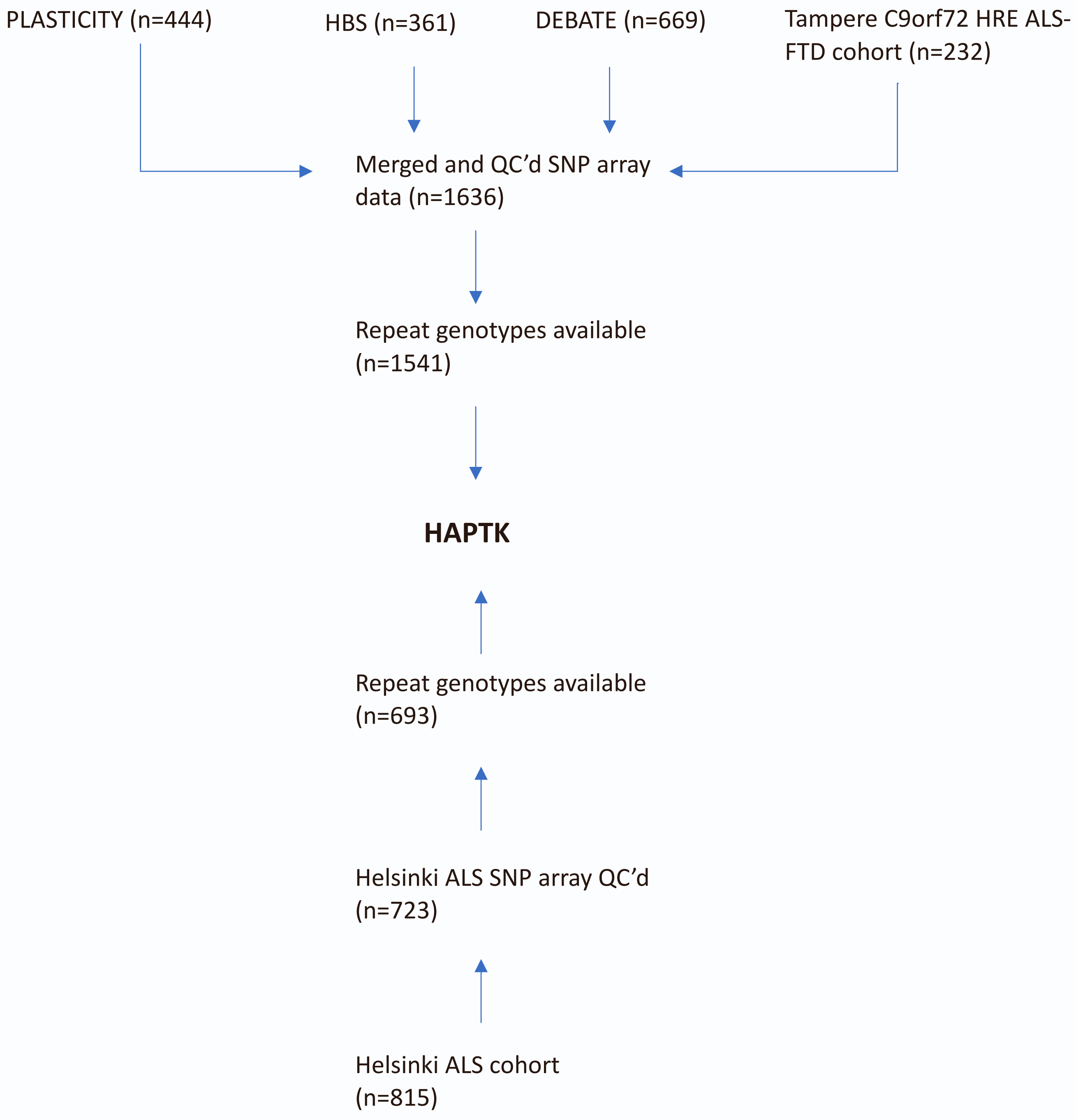

Figure S1. A chart of the study
Illumina GSA v2-3 chip cohorts on the top and the FinnGen Affymetrix Axiom Custom SNP array Helsinki ALS samples on the bottom. The number of samples per different repeat genotype group can be found in Tables S4 and S5.

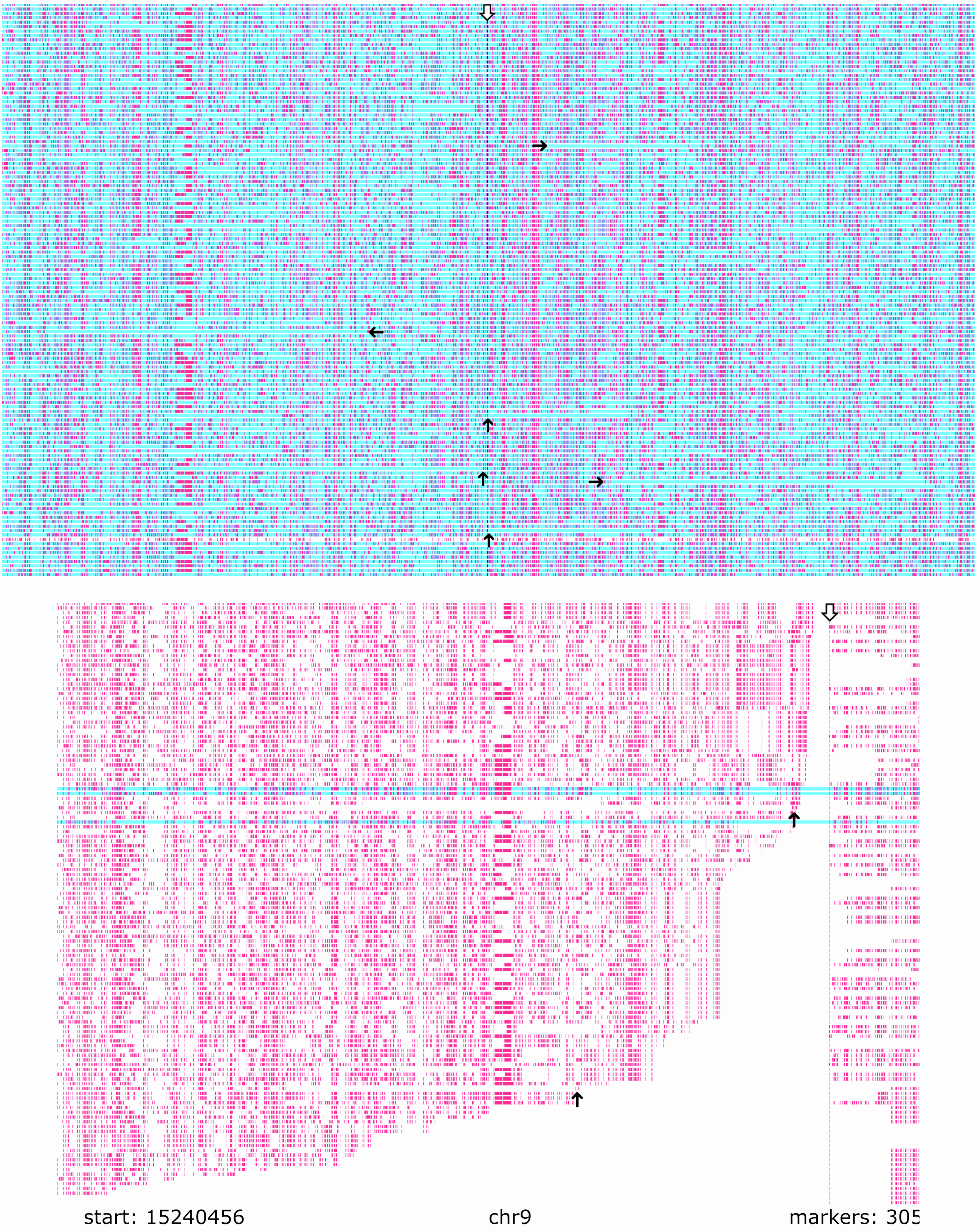

Figure S2. Switch-error and genotyping error evaluation based on the comparison graph
The white down-arrow on the top denotes the starting point of the algorithm (the C9orf72 HRE locus). The black up-arrows denote suspected genotyping errors or small switch-errors. Left and right arrows denote runs of ancestral sequence that are most likely due to a switch-error further away from the starting locus. The haplotypes tagged in blue were removed when selecting only for the longer ancestral sequence sharing haplotypes per sample.

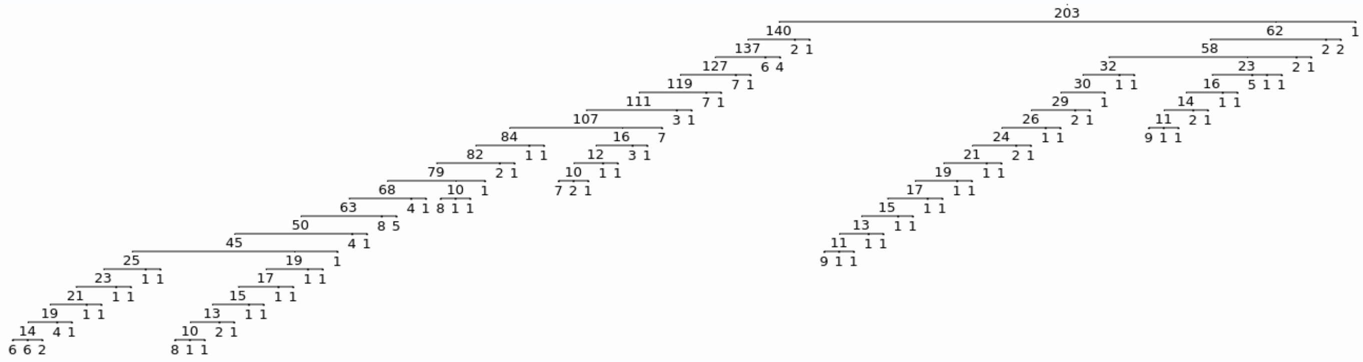

Figure S3. The Tampere ALS-FTD cohort bidirectional HST of HRE haplotypes

The cohort is genotyped on the Illumina GSA v3 SNP array. For visualization purposes the branching is cut after 10 samples.

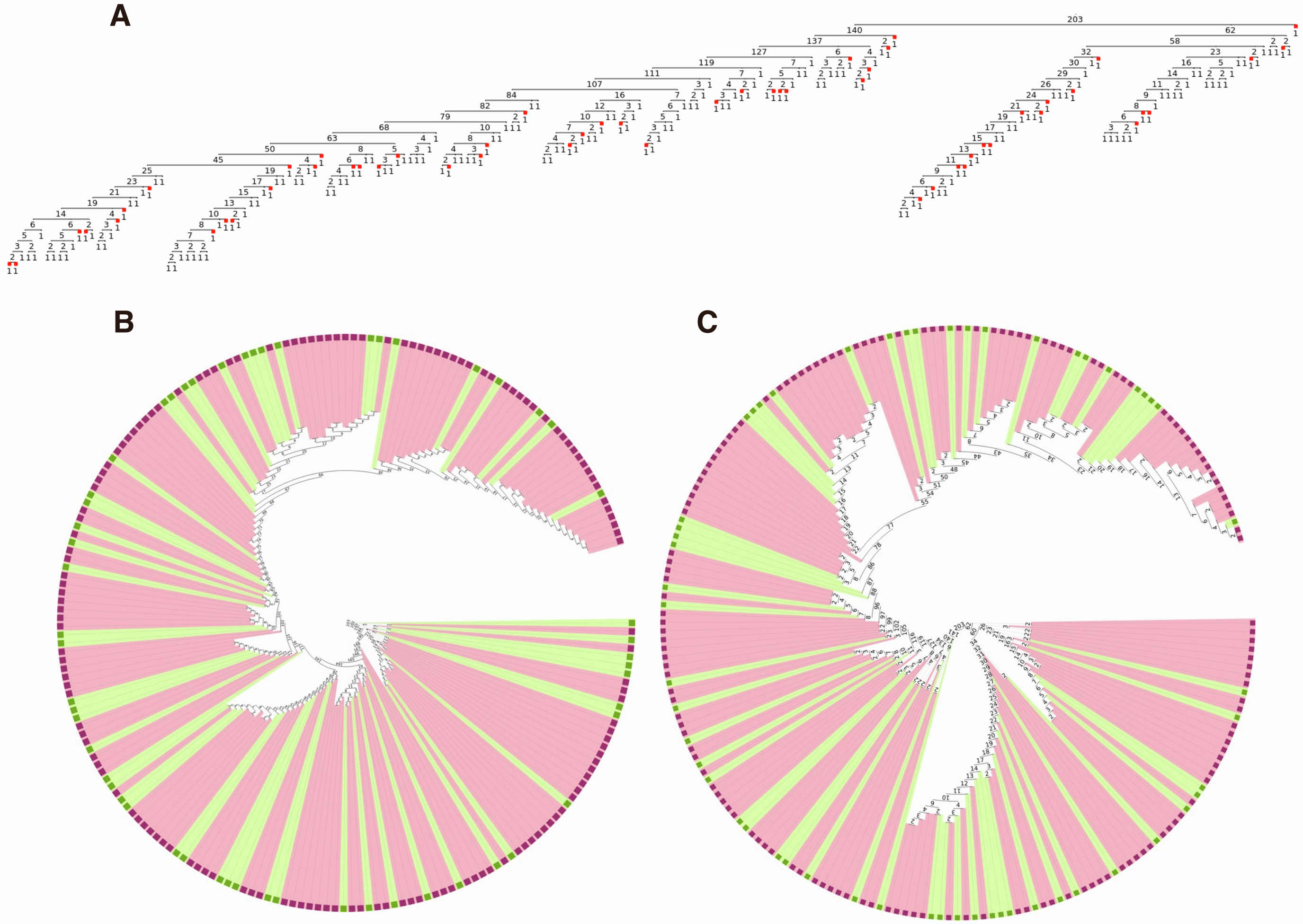

Figure S4. The Tampere ALS-FTD cohort bidirectional HST of HRE haplotypes with FTD samples tagged in red

(A, B, C) The FTD cases are relatively uniformly distributed along the tree and do not form their own subtrees.

(A) The bidirectional HST with FTD samples marked in red. No circular layout.

(B) The left side unidirectional HST with FTD samples marked in green.

(C) The right side unidirectional HST with FTD samples marked in green.

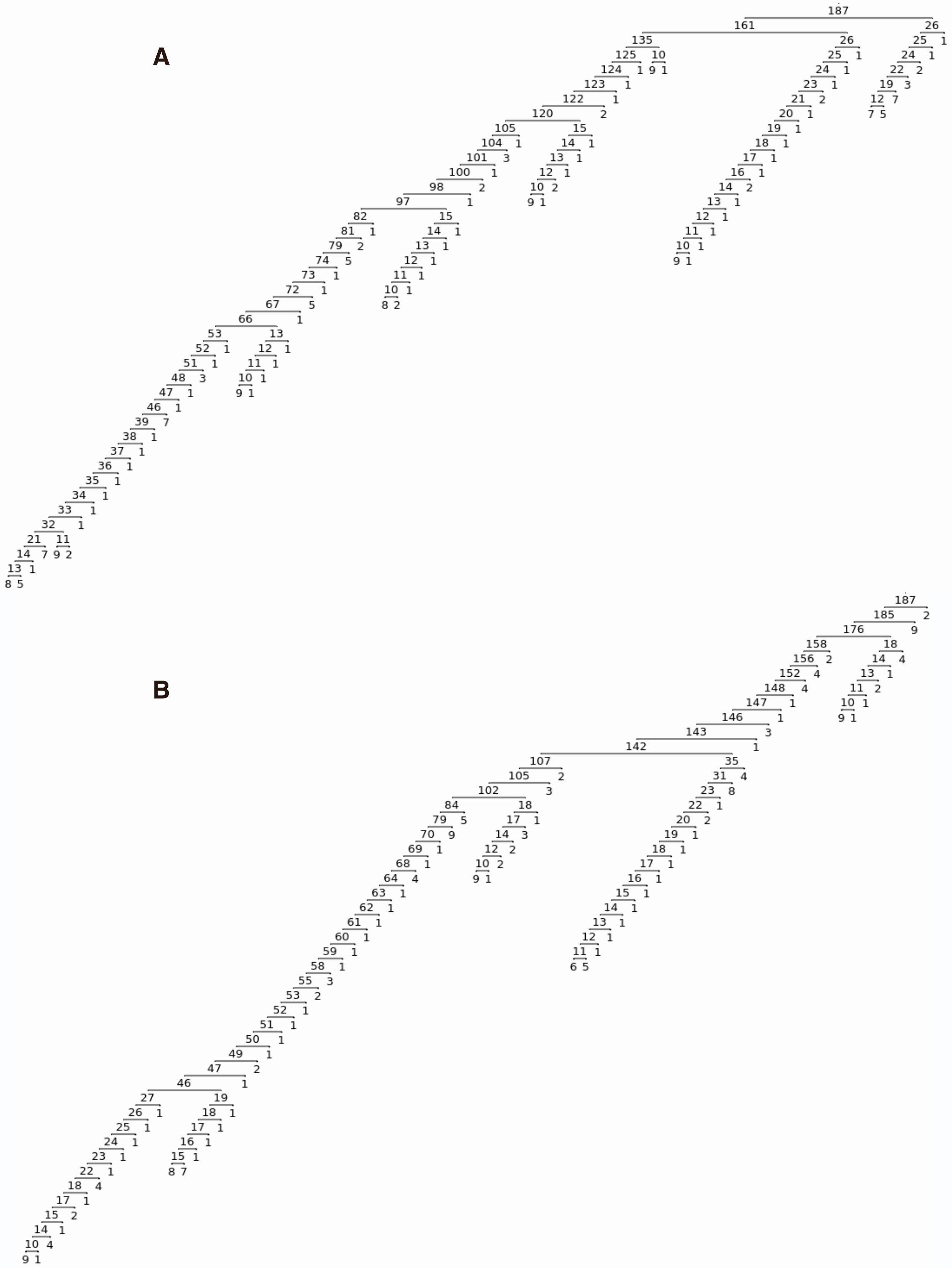

Figure S5. The Helsinki ALS cohort unidirectional HSTs of HRE haplotypes
(A) The right side HST **and (B)** the left side HST **(A and B)** The samples were genotyped with the FinnGen Affymetrix Axiom SNP array. For visualization purposes, the branching is cut after 10 samples.

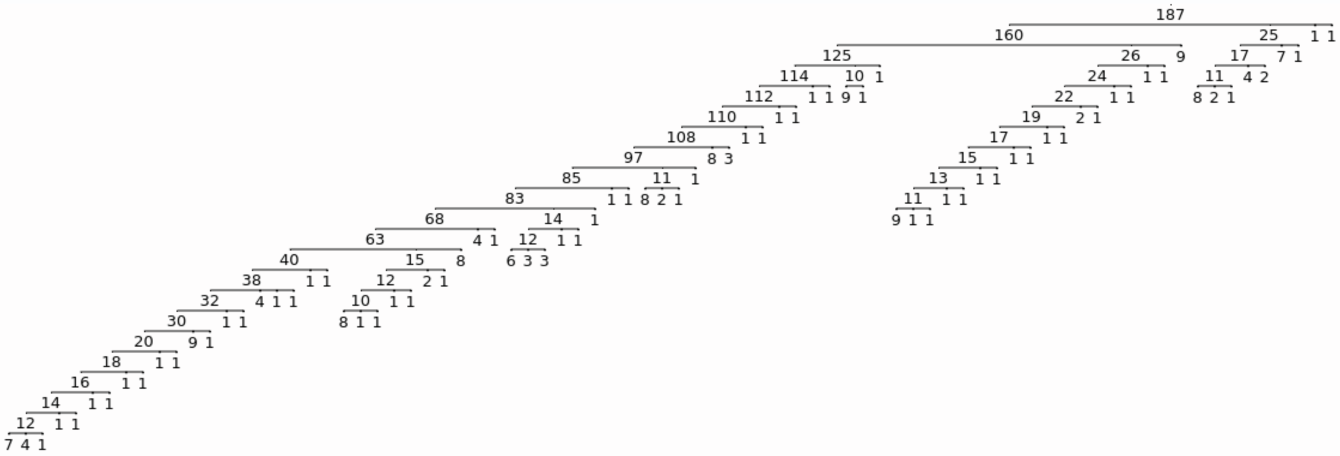

Figure S6. The Helsinki ALS cohort bidirectional HSTs of HRE haplotypes

Genotyped on the custom Affymetrix Axiom SNP array. For visualization purposes, the branching is cut after 10 samples.

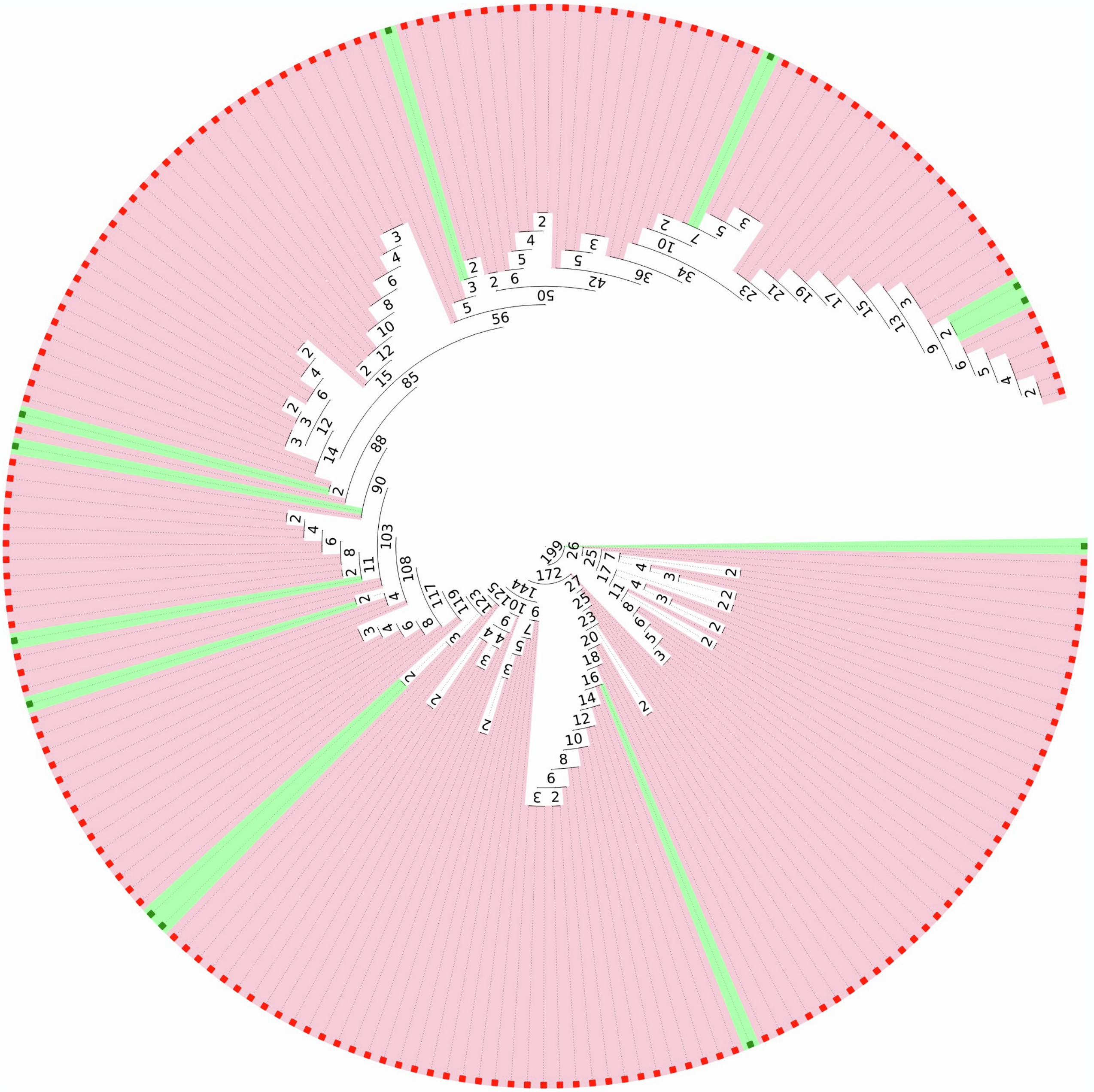

Figure S7. The Helsinki ALS cohort bidirectional HSTs of HREs mixed with ≥20 IAs.

The ≥20 repeat IAs are tagged in green.

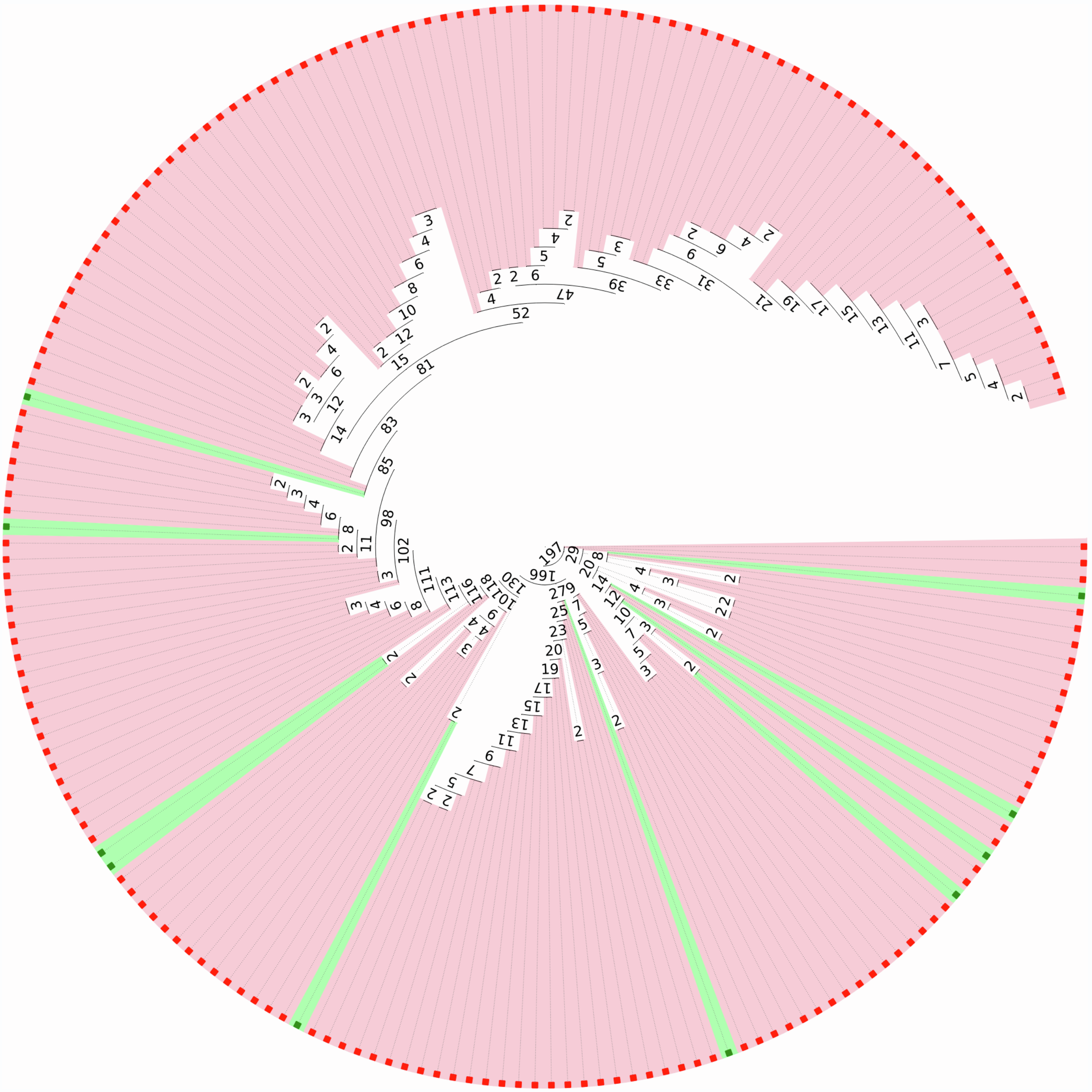

Figure S8. The Helsinki ALS cohort bidirectional HSTs of HREs mixed with 15-19 repeat IAs.
The ≥15 repeat IAs are tagged in green.

Supplemental Tables

Table S1. Genotype concordance between the AmplideX C9orf72 kit and RP-PCR

Table S2. Core HRE haplotype of the Tampere ALS-FTD cohort (Illumina GSA 24v3)

Table S3. Core HRE haplotype of the Helsinki ALS cohort (Affymetrix Axiom custom SNP array)

Table S4. Tampere ALS-FTD ancestral HRE haplotype

Table S5. Helsinki ALS ancestral HRE haplotype

Table S6: Tampere ALS-FTD cohort statistics in groups by GGGGCC repeat size

Table S7. Helsinki ALS cohort statistics in groups by GGGGCC repeat size

Table S8. Tampere ALS-FTD ancestral haplotype compared to Helsinki ALS ancestral haplotype

Table S9. Tampere ALS-FTD ancestral haplotype compared to Helsinki ALS ancestral haplotype with all variants shown

Supplemental Methods

1. Control cohorts

The Helsinki Businessmen study (HBS) included men with high socio-economic status who were born in 1919–1934 and in 2002–2003, 672 individuals who lived at home were randomly selected for analyses (DNA was available in 666).

The DEBATE study was originally a random sample of 4800 individuals from Helsinki out of whom 400 home-living individuals with stable cardiovascular disease were randomly selected for further studies in 2000 (DNA was available for 375). We excluded one individual diagnosed with ALS from these cohorts, which decreased the number of controls from the original 3142 to 3141.

The PLASTICITY cohort is an ongoing long-term follow-up of originally 1196 individuals born in 1971–74 in the Helsinki metropolitan area who had at least one predefined pre- or perinatal risk factor (e.g. low birth weight or Apgar score). 509 subjects were seen at age 40 (DNA available in 433).

2. Unidirectional haplotype sharing tree algorithm

The genotype matrix consists of biallelic phased data without missing genotypes and each sample has two rows of genotypes. The algorithm is run twice, once per direction.

Outline of the algorithm:
1. Select a starting marker from the genotype matrix by a coordinate, if the given coordinate has no designated variant, select the one closest to it.

2. Travel marker by marker towards a given direction from the starting marker.

3. When a contradictory genotype in a column of genotypes is found, assign samples into two new leaf nodes based on the genotypes. For biallelic data, two possible genotypes exist [0], [1].

4. Iterate all leaf nodes.

5. Repeat steps 2. and 3. starting from the last breakpoint and only in the context of the samples present in the currently iterated leaf node.

6. Continue until all leaf nodes have only a single sample left or sequencing data runs out

3. Bidirectional haplotype sharing tree algorithm

The genotype matrix consists of biallelic phased data without missing genotypes and each sample has two rows of genotypes. The algorithm is run once.

Outline of the algorithm:
1. Select a starting marker from the genotype matrix by a coordinate, if the given coordinate has no designated variant, select the one closest to it

2. Travel to the right from the starting marker until a contradictory genotype is found in the column.

3. Travel to the left from the starting marker until a contradictory genotype is found in the column.

4. When both sides present a contradictory genotype, assign samples to four possible genotype buckets: [0,1], [1,0] [0,0] [1,1]. For each bucket, add a new leaf node to the tree.

5. Iterate all leaf nodes.

6. Repeat steps 2. to 4. starting from the last breakpoint and only in the context of the samples present in the currently iterated leaf node.

7. Continue until all leaf nodes have only a single sample left or sequencing data runs out.
